# From Chew Counts to Intake Amounts: An Evaluation of Acoustic Sensing in Browsing Goats

**DOI:** 10.3390/s26020719

**Published:** 2026-01-21

**Authors:** Shilo Navon, Aharon Bellalu, Ezra Ben-Moshe, Hillary Voet, Eugene David Ungar

**Affiliations:** 1 Department of Natural Resources, Institute of Plant Sciences, Agricultural Research Organization (ARO), Volcani Center, 68 HaMaccabim Road, P.O. Box 15159, Rishon LeZion 7505101, Israel; shilo.navon@gmail.com (S.N.); aharonb@volcani.agri.gov.il (A.B.); bmezra@gmail.com (E.B.-M.); 2 Department of Environmental Economics and Management, The Robert H Smith Faculty of Agriculture, Food and Environment, The Hebrew University of Jerusalem, P.O. Box 12, Rehovot 7612001, Israel; hillary.voet@mail.huji.ac.il

**Keywords:** bite mass, carob leaves, chew–bites, chewing coefficient, comminution, inverse regression, jaw movements, satiety

## Abstract

Herbage intake by grazers and browsers is of fundamental importance to agricultural ecosystems worldwide but is also notoriously difficult to quantify. The intake process is mediated by herbage comminution in the mouth. The attendant chew actions generate sound bursts that can be detected acoustically and analyzed to help elucidate the entire process. Goats consuming a single plant species were acoustically monitored in order to (i) determine the sensitivity of the chewing effort to the large variation in bite mass and satiety level and (ii) estimate how well the amount of herbage consumed can be predicted by counting chews. Experiments used hand-constructed patches containing bite-sized carob (*Ceratonia siliqua* L.) leaflets of a pre-determined mass that were presented to six goats, individually, with acoustic sensors attached to their horns. Experiment 1 determined the chewing effort and the sequence of bites and chews for three bite masses across five levels of total intake. Experiment 2 determined the chewing effort and the chew sequence at three levels of satiety, achieved by control of the feeding regime, using a single bite mass across three levels of total intake. In Experiment 1, the global chewing coefficient was ≈4 chews g^−1^ fresh mass ingested (≈10 chews g^−1^ dry matter). For an individual animal, the chewing coefficient was fairly stable, being influenced mildly by bite mass, but the variation between animals was large. In Experiment 2, the chewing coefficient was again fairly stable in an individual animal, although the chewing effort was slightly elevated at low satiety. At the population level, and for the most relevant range of intake levels, inverse regression of the pooled data from both experiments estimated the two-sided 95% confidence interval of the predicted intake of carob leaves to be <10% of the predicted value. If chewing coefficients can be estimated locally, usefully precise intake predictions should be attainable for the tested vegetation. These results are promising for the future potential of acoustic monitoring, although significant challenges remain.

## 1. Introduction

Acoustic monitoring of animals during grazing is a promising sensor technology that has emerged in recent decades, with the potential to become an important tool for grazing research, as well as grazing management [1,2]. Technically, it entails the detection of the sound bursts generated by the bite and/or chew actions of individual animals when they graze or browse vegetation and the extraction of information from the signal. It has been demonstrated in cattle (e.g., [3,4]), sheep (e.g., [5,6]), and goats [7]. No particular type of microphone or location on the animal’s head is required, although contact with the head is essential for detecting chews well. When dealing with relatively short recordings, as in the present study, information can be extracted manually (i.e., aurally) from the acoustic signal. Importantly, no calibration or validation is required to determine what the animal did; under good recording conditions, the sounds are unmistakable, and their *identification* is definitive and essentially error-free. The *classification* of the sound bursts can achieve similar accuracy as well, depending somewhat on the vegetation and the individual animal. In studies that obtain much longer recordings than those conducted here, possibly spanning many hours or even days, it becomes essential to sequence jaw movements algorithmically (e.g., [8,9,10], which is likely to incur a cost in terms of accuracy [11].

The successful development of this sensor technology certainly depends on overcoming non-trivial technical hurdles, but the *motivation* to invest that effort depends on the value of the information it can provide. The simplest and most basic datum is the timeline of ingestion-related sound bursts, as point events. Beyond providing the rate of jaw movement at different temporal scales, this timeline can furnish a penetrating profile of how the animal interacts with its forage environment [7,12]. Somewhat more challengingly, the sound bursts can be classified into the three types of jaw movement of interest—pure bites, pure chews, and chew–bites (the latter known to be prevalent in cattle)—which enables the bite rate and chew rate to be quantified [13]. Most challengingly and *not* attempted here, analytic tools can be used to extract features of individual bite sounds and/or chew sounds that correlate with the bite mass. Several studies involving cattle or sheep have demonstrated relatively good correlations between the chewing energy and intake (e.g., [6,13,14]).

Intake is certainly the datum of highest value; however, addressing it in this very direct way entails relatively complex analysis in the frequency domain [15], and even then, it is uncertain how well the results will generalize across devices and forage environments. Starting simply, we consider to what extent intake can be based solely on the timeline of classified jaw movement events. There are two approaches: via bites or via chews.

Traditionally, the only way to study the ingestive behavior of grazing animals has been by direct visual observation. This enables bites, but not chews, to be counted with reliability, and, naturally, a bite-centric approach developed, which was focused on bite rate (e.g., [16,17,18]). However, to convert bites to intake requires an estimate of the bite mass, which is fairly intractable empirically. One way of addressing this problem, at least for herbaceous vegetation, has been to define the bite dimensions as functions of basic sward characteristics that *can* be measured. The bite mass then follows from the bite dimensions and herbage bulk density [19]. Hand-constructed swards played a major role in elucidating these functions (e.g., [20,21,22]), but extrapolating the findings to natural forage environments is difficult. Furthermore, the bite-dimensions approach is less relevant where plant structure and morphology predefine the bites on offer, as is often the case with woody species [23].

The chew-centric approach leverages the self-evident facts that (i) chewing is an unavoidable requirement of ingestion in herbivorous mammals (among other taxa), and (ii) the amount of chewing must be in some positive monotonic relationship with the amount of vegetation ingested. Conceptually at least, if not from a formal statistical perspective, the amount of chewing can be defined in terms of the chews performed per unit mass ingested, and the intake can be predicted from the ratio of the chew count to this “chewing coefficient”. However, this raises questions as to how variable such a coefficient might be and what primary factors might influence it. The present study sought to address these questions, also using hand-constructed swards of a kind, in order to evaluate the utility of the chew-based approach to intake prediction.

The conservatism of the animal’s chewing effort can be defined with respect to three axes. The first encompasses the type and even species of the vegetation consumed, including the part of the plant removed and its physiological state. This axis was *not* examined here, and the vegetation was standardized, although the same methodology could be applied more broadly. The second and third axes, which *were* examined here, are derived from a closer examination of the mechanics of the ingestive process, and differentiation between what we term here as chewing effectiveness and chewing requirements. The chewing effectiveness, as used by Pérez-Barbería and Gordon [24], relates to the effectiveness of a chew in comminution (crushing, grinding, and mashing) of the herbage in the mouth. The chewing requirement relates to how small the particles in the mouth must be to trigger swallowing. Short of using esophageal fistulated animals (which we did not), these factors are not directly measurable, and their role can only be inferred. Nevertheless, intake prediction from the chew number depends on the stability of *both* these factors. The more stable they are, the tighter is the expected coupling between the intake and chew number.

There are reasons to expect a degree of stability. Browsing activity in herbivores is subject to the opposing selective pressures of meeting a strict nutritional requirement while avoiding predation. Evolutionary solutions to this trade-off are expected to favor browsing strategies that meet nutritional requirements in the shortest possible time. Optimized ingestive behavior is a central component of such a solution and necessarily involves maintaining high and as stable as possible chewing effectiveness. Such performance can be achieved by processing a large and consistent amount of feed per chew. Nevertheless, real-life factors such as vegetation patchiness, fine-scale availability, and most importantly, bite sizes and their distribution within the stream of jaw movements may introduce inefficiencies of unknown magnitude into this otherwise assumed optimized system. We hypothesized that the effectiveness of a chew action depends on the level of foliage loading in the mouth. This would be of consequence if animals processed bites one at a time, or if the foliage loading depended on the bite mass: we might expect a low bite mass to depress the chewing effectiveness because the limiting factor is the rate at which foliage is fed into the mouth by bite actions. At a high bite mass, the animal is expected to operate at the highest chewing effectiveness because the breakdown of the herbage is the limiting factor. Based on this reasoning, the bite mass was the key factor examined in relation to the chewing effectiveness (Experiment 1).

Regarding the chewing *requirement*, we hypothesized that the factor most likely to influence it was the level of satiety. Our expectation was that hungry animals (low satiety) invest less effort in chewing than sated ones to more quickly alleviate hunger. The diminished chewing effort would be enabled by permitting larger particle sizes to be swallowed. Such a reduction in the chewing requirement is plausible, given that rumination chewing could compensate [24]. Satiety was therefore the key factor examined in relation to the chewing requirement (Experiment 2).

The ability to estimate the intake on the basis of chewing is especially useful in environments where indirect estimation by interval sampling of the vegetation is most difficult, such as those in which woody vegetation forms a substantial portion of the diet. This is commonly the case for goats reared in Mediterranean regions. As the model plant, we selected a common woody species of such regions, whose morphology lends itself well to the hand-constructed foraging arena. We tested the response of the chewing effectiveness to large variations in bite mass in short eating sessions and the response of the chewing requirement to the level of satiety, for goats consuming the foliage of a woody species. The broader objective was to assess how well we can predict the herbage intake from the number of chews performed, at much larger time scales. Two interpretations of the chewing coefficient were tested for their implications as to how the concept might be applied. We are not aware of other studies of goat herbivory (albeit somewhat simulated) using acoustic monitoring in conjunction with precisely controlled bite mass and intake (the study by Wang et al. [15] used acoustically-monitored goats, but they were hand-fed). Theoretical analyses of herbage processing by herbivores in the mouth have been reported [25,26], but this appears to be the first empirical test of alternative models. The results should help evaluate the potential utility of acoustic monitoring as a sensing technology for grazing animals.

## 2. Materials and Methods

### 2.1. Overview

Two experiments were conducted using a methodology that combined acoustic monitoring to count the chews precisely and hand-constructed presentation of the vegetation to precisely control the bite mass and total intake. Experiment 1 tested the response of chewing effectiveness to large variations in bite mass, while Experiment 2 tested the response of the chewing requirement to the satiety level. Both experiments used the same model vegetation and the same six individual animals, although the protocol used in Experiment 1 enabled a more exhaustive analysis. As a point of terminology, “bite” and “chew”, as used here, include both pure actions and chew—bite actions; the terms “pure bite” and “pure chew” exclude chew–bites.

### 2.2. Experimental Animals and Their Management

The experimental animals were drawn from a herd of ≈120 dairy goats of the Damascus (Shami) breed (*Capra hircus* L.) owned by Ramat Hanadiv Nature Park in northern Israel (https://www.ramat-hanadiv.org.il/en/ (accessed on 11 January 2026). The herd housing and facilities were located near the park’s center. Although concentrate feed was provided at milking, the bulk of the animals’ nutritional requirements was met by foraging. To this end, the herd was shepherded daily along foraging excursions that exploited the surrounding Mediterranean garrigue vegetation, as an integral part of the park’s management.

Once designated as such, the experimental animals continued to be milked together with the rest of the herd throughout the experimental periods. They joined the herd on its daily foraging excursion on all but the training and observation (test) days. On those days, the animals were separated to an adjacent holding area before the herd departed the animal barn, and after the herd’s departure, the training and testing sessions were conducted in close proximity to the holding area. Basic information regarding the six experimental animals is provided in Supplementary Table S1. The incisor arcade width was surveyed (on 14 May 2012) across 30 similarly-aged animals in the herd. A tight distribution of values around an average of 31 mm was obtained. The incisor arcade width was not measured on the experimental animals to avoid additional stress.

### 2.3. Model Plant Species

The model plant species was the carob (*Ceratonia siliqua* L.), a dioecious evergreen tree of the family *Leguminosae* that is abundant in low warm habitats. Carob is an important forage source consumed readily by goats. All carob leaves were harvested from a cluster of female trees (Supplementary Figure S1A), located (for logistic convenience) near Karmei Yosef, Israel (31°50′54″ N 34°55′13″ E). The quantity of foliage required for either experiment exceeded the readily-accessible foliage supply of any one tree. The leaves of the carob tree are long and pinnate, with 6–10 leaflets (Supplementary Figure S1B), enabling relatively easy control over the bite mass by removing and/or trimming leaflets.

### 2.4. Preparation of Bite-Sized Units

The carob-tree foliage was always cut and prepared the day before its use. Small branches were cut from the tree, sprayed with water to suppress the moisture loss, placed in large plastic bags, and brought directly to the lab for preparation of the bite units. The target bite masses of 0.6, 1.2, and 2.4 g fresh mass corresponded fairly well to plant units having 1, 2, and 4 leaflets, respectively. The upper end of the range examined was based on the preliminary trials conducted in March and May of 2011 (with different goats) to refine and calibrate the methodology. The leaflets of these plant units were trimmed as needed, such that the entire plant unit was within 0.1 g of the target bite mass (determined by an electronic scale with a resolution of ±0.001 g). The prepared material was put immediately in plastic bags and stored overnight in a refrigerator at 4 °C. On the trial day, the bags were transferred to a cool box and removed as needed for the construction of hand-constructed patches. The material for an individual test session was removed from the cool box approximately 30 min in advance to allow equilibration with the ambient temperature. The moisture loss during this period was not a concern; based on ancillary measurements, carob leaves lost ≈0.05 of their fresh mass during three hours of air exposure under similar ambient conditions.

### 2.5. Hand-Constructed Patch

**Figure 1:**
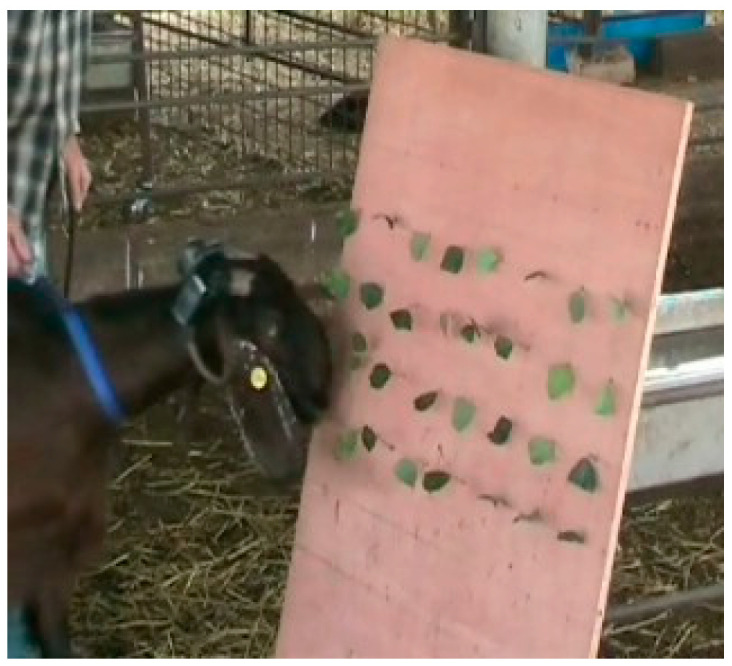
Video frame at the start of a test session in Experiment 1 showing the hand-constructed patch used to simulate foraging from a bush. In this example, four rows of eight holes were populated with one leaflet of 0.6 g fresh mass, for a total intake of 19.2 g fresh mass. One of the acoustic sensors is clearly visible on the animal’s right horn. The operator to the animal’s left holds a leash loosely.

The bite-sized units of carob leaves were organized, with the assistance of a baseboard, into a hand-constructed patch of foliage that served as a simplified bush when presented upright. The height of the vegetation was always above the animal’s withers, forcing an upright head position typical of browsing (Figure 1). The precise control of the bite mass and total intake was achieved by populating the board with the required number of single-bite-sized units of 0.6, 1.2, or 2.4 g fresh mass, spaced sufficiently apart to force the animal to expend exactly one bite action to harvest each unit. The final configuration described here was the outcome of periodic testing and refinement over a two-year period.

The baseboards used in both experiments comprised a plywood board (80 × 120 × 1.5 cm) into which holes were drilled to form an upright rectangular lattice of four rows of eight holes, spaced 10 cm within rows and 16 cm between rows. The holes traversed the entire thickness of the board at a slope of 35–40° in relation to the board surface plane. The hole diameter of 3 mm was just sufficient to accommodate the thin stem-like base (rachis) of one plant element. The rachis was inserted into the hole without clamping or fixing. This angled arrangement was found to provide sufficient resistance to detachment, by virtue of the friction between the rachis and the inner surface of the hole, to evince a fairly normal head motion associated with a true bite, while still ensuring that the plant element was pulled out in its entirety to obtain the intended bite mass. Even though these bite-like actions lack the severance component of a true bite action, for brevity, we refer to them as “bites” nevertheless.

### 2.6. Acoustic Monitoring

Both experiments used acoustic monitoring to derive the chew count that accompanied the consumption of the hand-constructed patches. For sound detection, a piezoelectric microphone was used (Model WCP-55; Cherub Technology Co., Nanshan, China). This contact microphone is effective at screening out external noises, which is a major concern in an active animal barn. The microphone was connected to a Sansa Clip+ MP3 Player (SanDisk, Milpitas, CA, USA) that was modified to receive sound input from the external microphone. Details of the modifications made to the recording device with respect to the microphone are given in [12]. The recordings were stored on internal memory in .wav format and at a sampling frequency of 32 kHz. At deployment, following initialization, the recording device was placed inside a customized protective metal casing that had an aperture for the cable leading to the external microphone (A. Braun Metals, Tel-Aviv, Israel). The entire assembly was then attached to the left horn of the goat being tested, with the microphone’s cushioned diaphragm pressed flush against the smoothest face of the animals’ horn. At the start and termination of every recording session, a set of taps were recorded at a precisely known time. These reference points were used later to change the nominal sampling frequency in the .wav file header to a more precise value. This was necessary in order to correctly splice the soundtrack onto video (see Section 2.7) and to obtain more accurate estimates of the inter-event interval.

### 2.7. Acoustically Augmented Video

In the first and more complex experiment the entire proceedings were filmed by video camcorder (Model MV900; Canon, Tokyo, Japan) as a definitive record of the sequence of animals and patch treatments tested on each day of the trials. The camcorder was positioned on a tripod to the rear and opposite side of where the animal would stand when consuming the patch, with the entire animal and board within the frame.

To extract higher value from the video, a microphone transmitter–receiver system (Model RB-50; Elkat Security Engineering Ltd., Tel Aviv, Israel) provided the soundtrack of the video. This also served as a backup to the primary acoustic sensor. The microphone transmitter component was attached to the right horn of the test animal, and the receiver was connected to the audio input jack of the video camera. We used a subminiature condenser Lavalier microphone (Shure Incorporated, Niles, IL, USA, model WL93), which yields a stronger signal than a piezoelectric microphone on the opposing horn of the animal, but it collects all the ambient noise. The microphone was connected via a short cable to the battery-powered and crystal-controlled FM wireless transmitter (dimensions 38 × 28 × 10 mm) operating at 173.225 MHz. A standard PP3 9V battery was connected externally. The similarly-powered FM receiver (dimensions 108 × 55 × 27 mm) provided headphone and auxiliary audio outputs. The audio output from the receiver was connected to the audio input of the video camcorder.

There were methodological differences between Experiments 1 and 2 that changed how the acoustic signals were processed. For this reason, the methodologies are elaborated first, followed by processing of the acoustic signal in Section 2.11.

### 2.8. General Experimental Protocol

Prior to each foraging session—in which one hand-constructed patch would be depleted by a single animal—the animal number, treatment bite mass, and treatment bite number were announced and captured on the video soundtrack. The left horn, bearing the contact microphone, was tapped audibly at the start and termination of each set of sessions in order to synchronize the two recordings. The taps propagated to the opposing horn and were detected by the wireless system. With the assistance of a collar and leash, the target animal would be led calmly along the short distance from the holding area to the upright foraging board, approaching diagonally for the best video camera angle. The animal was allowed to commence depletion without delay. After complete depletion of the board, the animal was restrained in position until the chewing terminated, and the last bolus was swallowed. The animal was then led away while the next patch was prepared. The absence of jaw activity was verified in the moments prior to all sessions to assure an empty mouth at commencement.

### 2.9. Experiment 1: Bite Mass

#### 2.9.1. Experimental Design of Experiment 1

To estimate the response of the chewing effort to bite mass, a full-factorial experimental design was used. The first factor, bite mass, was tested at three levels: 0.6, 1.2, and 2.4 g fresh mass (for brevity, units of herbage mass are the fresh mass throughout, unless otherwise stated). The second factor was the total intake, for which five levels were examined within each bite mass: 2.4, 4.8, 9.6, 14.4, and 19.2 g, facilitating a regression-based approach for estimation of the chewing coefficient. The 15 treatment combinations were achieved by regulating the bite number, i.e., the number of holes populated in the hand-constructed patch, as shown in Table 1. In total, the 15 treatment combinations amounted to the consumption of ≈150 g of carob foliage per animal (excluding repeated boards). On the measurement days, animals received their usual ≈500 g of high-quality supplementary feed during milking. Water and roughage feed were available in the group holding pen, in which they were separated prior to the main herd departing the animal barn.

#### 2.9.2. Detailed Protocol of Experiment 1

The timeline for Experiment 1 began in January 2012 with the selection of eight primi- and multiparous goats, including two alternates. Preference was given to early-lactation goats of easy temperament, similar age, and live mass, with horns suitable for the acoustic monitoring equipment. The constraints of temperament, kidding window, and horn configuration and the eventual need to use both alternates precluded high uniformity in age and live weight (Supplementary Table S1).

Trial runs to familiarize the animals with the experimental protocol were conducted on 26 January, 22 February, and 3 April 2012. This included the attachment of acoustic sensors to the horns and depletion of the hand-constructed patches. Goats were again familiarized with the protocol the day before and on the morning of the day they were tested. The experiment itself was conducted on three dates: 24 April, 3 May, and 8 May 2012, with all treatments tested on two animals on each date (goats 775 and 727; 830 and 76; and 712 and 724; respectively). Each goat was allowed to deplete a training patch as a final reinforcement and was then offered the 15 treatment combinations in succession, in fully randomized order, with short breaks to refresh the hand-constructed patches. The individuals to be tested were drawn from and returned to the holding pen, where water and roughage were available.

In the course of the experiments, irregularities occurred that triggered disqualification of a session and its subsequent repetition. The primary irregularities were (i) a head movement of the goat causing a plant unit to dislodge from the board and fall to the ground; (ii) a leaflet hanging from the side of the mouth, as part of a larger bite-sized unit, becoming detached and falling to the ground; (iii) severance of part of a plant unit in the course of a bite action, leaving part of it in the hole; and (iv) more-than-momentary and atypical distraction of the animal’s attention or interruption of the normative rhythm of jaw movements.

### 2.10. Experiment 2: Satiety Level

#### 2.10.1. Experimental Design of Experiment 2

The effect of satiety on the chewing effort was tested at three sequential satiety levels (low, medium, and high). The satiety was controlled by metering the supplementary feed over the course of the test day. At each satiety level, the chewing effort was estimated at three levels of intake: 9.6, 14.4, 19.2 g, achieved by the consumption of 4, 6, and 8 bites, respectively, of constant mass, 2.4 g. The treatments were replicated over the same six goats that participated in Experiment 1. The goat sequence and intake level within each goat were both randomized.

#### 2.10.2. Detailed Protocol of Experiment 2

In place of the single holding pen used in Experiment 1 for the entire group of experimental animals, individual-animal pens were erected in the same location in Experiment 2. This enabled the precise control of the daily intake at the individual-animal level. The 1.1 m tall sides of the pens were constructed of rigid metal wire mesh with shared sides and provided an area of 1.2 × 2.5 m for each animal (Supplementary Figure S2). Each pen contained a bucket with clean water and a plastic basin into which controlled amounts of feed were provided. The feed mix contained small quantities of chopped hay, concentrates, green Morus leaves (*Morus alba* L.), and carob leaves, which was intended to maintain a similar motivation to consume the carob leaves across satiety levels.

On training and observation days, the experimental animals were not given supplementary feed during milking. At ≈10:00 h, shortly before the low-satiety treatment set, the six animals were allowed to consume a small quantity (10–20 g fresh mass) of carob leaves during training sessions. This was followed by each goat in turn depleting the three treatments in randomized order. Medium satiety was similarly tested at 12:30 h, after the animals had consumed, on average, 570 g concentrates, 300 g hay, 30 g carob leaves, and 10 g Morus leaves. High satiety was tested at 15:20 h, after the animals had consumed cumulatively that day, on average, 1140 g concentrates, 624 g hay, 50 g carob leaves, and 12 g Morus leaves. A complete training run of the experiment was conducted on 18 June 2012, and the experiment itself was conducted the following day.

### 2.11. Counting and Sequencing of Chews and Bites

In Experiment 1, the total number of chews performed by a goat while consuming the foliage offered by a hand-constructed patch was determined by viewing and listening to an edited version of the video record for which one of the stereo channels was substituted with the soundtrack generated by the vibration microphone (after correction of the sampling frequency). The primary determination of the occurrence of a chew was based on the latter signal, supported by the condenser-microphone signal and the video image. There was one instance in Experiment 1 of poor signal quality from the vibration-based acoustic sensor (goat 727), and aural sequencing was based on the signal of the condenser microphone. The aural sequencing was performed independently by two of the investigators (S.N. and E.D.U.), and the few differences that arose were re-examined and reconciled.

Additionally, in both experiments, the sequencing was repeated aurally by one investigator (E.D.U.) in conjunction with event-marking tools in Sonic Visualiser v2.1 [27] in order to obtain the precise timing of individual chew actions. There was no risk of mistakenly registering bite actions as chews because there was no tearing or detachment of vegetation that ordinarily generates the distinctive sound burst associated with a bite; this ensured that all sound bursts were generated by chew actions (pure chews or chew–bites).

In Experiment 1, with the assistance of the video recordings, the precise number of “bites” removed, as defined by the treatment, was interleaved into the timeline of chews, in correct sequential order. Pure bites were marked at an arbitrary timestamp within the possible range. Jaw movements that performed both a bite action (verified from the video image) and a chew action (verified from the synchronized acoustic signal) in one duty cycle were marked as chew–bites in the sequence. Chew–bites were counted as both a bite and a chew action. The three layers of time-flags for the three types of jaw movement were exported and interleaved chronologically. The timestamps of pure bites were not used in any calculation of a time interval.

### 2.12. Herbage Moisture Content and Chemical Analysis

The dry matter content of the carob foliage was determined in the course of the experiments. The foliage was clipped at ≈06:00 h and weighed soon thereafter, dried at 68 °C for four days, and reweighed to determine the dry matter content and its variability. The sampled foliage comprised leaflets of fresh mass 0.6 g and entire-leaf samples of fresh mass 2.4 g (total *n* = 48 and 22, respectively). Additional foliage was sampled in the course of the experiments for the purposes of chemical and nutritional analysis by NIRS (near infrared reflectance spectroscopy). The leaves were dried in a forced-air oven at 55 °C for four days and then ground to pass through a 1 mm mesh. The material was pooled and mixed by experiment, and vials containing 5 g of material were subjected to NIRS analysis. Technical specifications of the system, as well as development of the calibration equations for the tanniferous browse foliage that were applied to the carob foliage scans, are provided in Landau et al. [28] and Glasser et al. [29].

### 2.13. Mechanistic Exploration of the Data

All data exploration and analysis were conducted using JMP Pro 18.0.2 software (SAS Institute, Cary, NC, USA). Four graphical devices were employed to provide a more mechanistic understanding of the data. The first was based on the event-based plots used by Ungar and Horn [7] and Ungar and Nevo [12]. As the precise timing of pure bites was unknown, the jaw movement rhythm was described in terms of the interval between the nearest chew actions. The inter-chew time interval was always calculated to the previous chew action, which could be the preceding event in the jaw movement stream or be interceded by one or more pure bites, which are ignored in the interval calculation. The event-based plots arranged all jaw movement events sequentially along the *x*-axis and show the following on the *y*-axis: (i) the inter-chew time interval for all chew actions in the sequence; or (ii) an arbitrary out-of-range value of 0.1 s to mark the relative position of all pure bites in the sequence. The points were color-coded to distinguish consecutive chews from those preceded by one or more pure bites. Second, we examined the frequency distribution of the inter-chew intervals for consecutive and non-consecutive chews. Third, we constructed staircase diagrams showing the progression of the total number of chew actions performed versus either the total number of bite actions performed or the intake. With each pure chew performed, the line advances vertically one unit; with each pure bite, the line advances horizontally by one unit or by the mass of one bite. Chew–bites advance in both directions simultaneously and, therefore, appear as diagonal lines. Fourth, we examined the frequency distribution of the length of runs of consecutive bites and of consecutive chews across treatments.

For Experiment 1, the data exploration phase included linear regression between the chew number (*y*) and intake (*x*) for each combination of goat and bite mass, noting the slope of the regression line, the intercept, their significance levels, and the goodness of fit (*r*^2^).

### 2.14. Statistical Analysis

Four considerations shaped the formal analysis to test the response of the chewing effort to the wide variation in bite mass in Experiment 1: first, whether the approach should be, at its core, the regression of chews on intake or the analysis of the chewing coefficient; second, whether the bite mass and intake should be (independently) defined as nominal or continuous variables (when using bite mass as nominal, significant linearity would be tested using an appropriate contrast); third, how best to account for an expected multiplicative effect of the bite mass on the chewing effort as opposed to an additive one; fourth, how the heteroscedasticity that became apparent in the data should be handled.

The preferred model used the chewing coefficient as the dependent variable, and both bite mass and intake were defined as continuous variables. The multiplicative effect of the bite mass was enabled by using the chewing coefficient as the dependent variable, which also reduced the heteroscedasticity but did not eliminate it. The implications of that were tested. The non-linearity in responses to the continuous factors was explored using quadratic terms or log transformation. The independent terms in the final mixed-model analysis of variance were goat (random), bite mass (continuous), and log of intake (continuous).

For Experiment 2, both satiety (3 levels) and intake (3 levels) were treated as nominal variables. The mixed-model analysis of variance of the chewing coefficient contained the terms goat (random), intake (nominal), and satiety level (nominal).

In using the chewing coefficient as a predictive tool, the bite mass and satiety would most probably not be known, and at the barest minimum at the population level, the intake would be predicted from chew counts only. To this end, linear regression of the pooled dataset of both experiments together was performed, and inverse regression was used to estimate the quality of the prediction. Incorporating information about the target animals or individualized estimation of the chewing coefficient are also addressed in this context.

## 3. Results

### 3.1. Carob Foliage Characteristics

The mean dry matter content (DMC; dry mass relative to fresh mass) of carob leaflets was 0.42; the mean foliage moisture content (FMC; mass of water relative to dry mass) was 1.42. Variability was low: the standard deviations of DMC and FMC were 0.04 and 0.26, respectively. Based on a DMC of 0.42, the fresh mass values of 0.6, 1.2, and 2.4 g used in the hand-constructed patches were approximately equivalent to 0.25, 0.5, and 1 g DM, respectively. Chemical analysis of the carob foliage based on the NIRS analysis is summarized in Supplementary Table S2.

### 3.2. Animal Welfare

For the most part, the experimental animals tolerated well all aspects of their handling in the course of the two experiments. The animals displayed no adverse responses to the acoustic equipment on each horn, such as head shaking or elevated irritability, and appeared indifferent to their presence. In general, the animals remained calm while being led to the hand-constructed patches and consumed the vegetation eagerly in a single bout, sometimes with momentary pauses. When that was not the case, the session would be disqualified and repeated. In Experiment 1, a complete set of hand-constructed patches, with any repeats, typically lasted ≈25 min for each animal. Despite initial screening for calmness, two animals proved to be skittish during the test sessions, and they were replaced by the pre-designated and trained alternates. The individual penning used in Experiment 2 did not result in any detectable behavioral changes that might be indicative of elevated stress or irritability. In Experiment 2, testing of a set of three hand-constructed patches typically lasted ≈5 min.

### 3.3. Acoustic Signal Waveform

**Figure 2:**
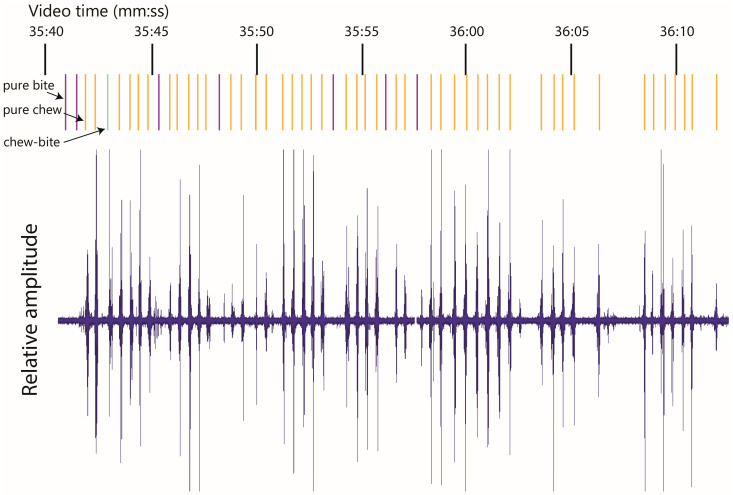
An example of the acoustic signal time-domain amplitude waveform generated in the course of one test session in Experiment 1 (goat 712; board 23; bite mass 1.2 g; total bites 8; total intake 9.6 g). For pure bites, lines indicate their precise *relative position* in the jaw movement sequence.

An example of the acoustic signal waveform generated in the course of a test session (in Experiment 1) is shown in Figure 2. It was clear, both aurally and from the waveform, that not all chews are equal in their sound intensity, with a small minority being relatively faint. Nevertheless, all chews were registered equally. Figure 2 also shows the positioning of all jaw movements performed; their timings are precise (small error relative to the inter-event interval) for pure chews and chew–bites (centered on the chew sound burst), and only relative for pure bites.

### 3.4. Bite Mass Experiment

#### 3.4.1. Data Overview of Experiment 1

As an initial quantitative orientation, the total amount of vegetation consumed in the entire experiment was 904 g, and 3789 chew actions (pure chew or chew–bite) were performed in consuming it, yielding a coefficient of 4.2 chews g^−1^ intake. A total of 880 bite actions (pure bite or chew—bite) were performed, yielding a similar numeric ratio of 4.3, but with units of chews bite^−1^. However, there was a clearly stronger and more linear relationship between the chews performed and amount of herbage consumed than between the chews performed and number of bites performed (Supplementary Figure S3). Even so, there was considerable variation around the general trend line in chews versus intake, and its partitioning is examined in the formal analysis (Section 3.4.4). The total number of chew actions performed by an animal in order to consume a complete set of treatments ranged from 425 to 797, showing that the chewing effort was *not* well-conserved across individuals. The total net consumption time (based on the first and last chews of each session, which slightly underestimates the true value) was 2186 s, yielding an overall instantaneous intake rate of 24.8 g min^−1^ (≈9.9 g DM min^−1^). This value ranged from 22.2 to 25.9 g min^−1^ across five of the animals and was 31.8 g min^−1^ for the sixth.

**Figure 3:**
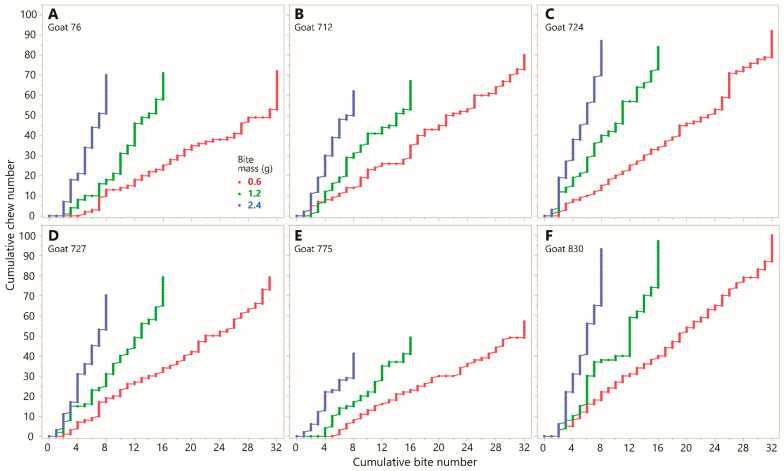
Chew versus bite staircase diagrams from Experiment 1 showing the chronological sequence of chew actions (*y*-axis) and bite actions (*x*-axis), expressed cumulatively from lower left to upper right, for three bite masses (indicated in panel (**A**)) at constant total intake (19.2 g). Each panel corresponds to one experimental animal. Chew–bites appear as diagonal increments. Scaling is uniform across panels but not isometric. (**A**) Goat 76; (**B**) Goat 712; (**C**) Goat 724; (**D**) Goat 727; (**E**) Goat 775; (**F**) Goat 830.

**Figure 4:**
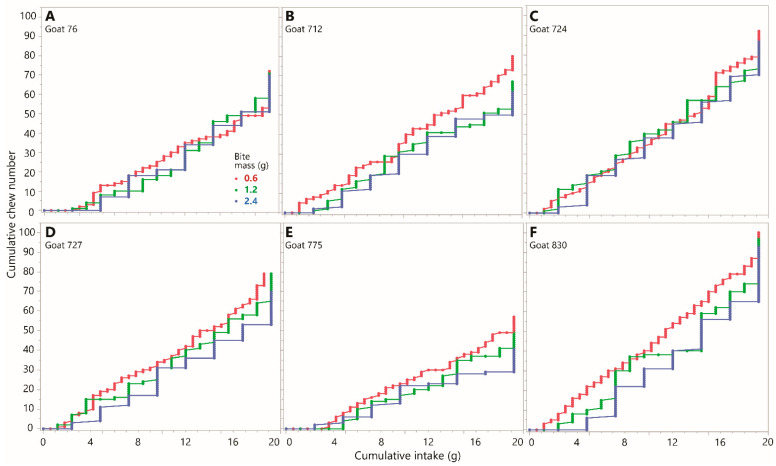
Chew versus intake staircase diagrams from Experiment 1 showing the chronological sequence of chew actions (*y*-axis) and bite actions converted to intake (*x*-axis), expressed cumulatively from lower left to upper right, for three bite masses (indicated in panel (**A**)) at constant total intake (19.2 g). Each panel corresponds to one experimental animal. Chew–bites appear as diagonal increments. Scaling is uniform across panels. (**A**) Goat 76; (**B**) Goat 712; (**C**) Goat 724; (**D**) Goat 727; (**E**) Goat 775; (**F**) Goat 830.

The primacy of the mass consumed over the number of bites removed in determining the number of chews performed is further supported by a comparison of selected staircase diagrams. These were constructed for the same intake (19.2 g; the maximum tested in the experiment) achieved using the three different bite masses of 0.6, 1.2, and 2.4 g. A comparison of chews versus bites (Figure 3) and chews versus intake (Figure 4) shows unequivocally that intake is a far better predictor of chewing requirements than is bite count. The formal analysis will test whether bite mass may have an influence on the overall slope of the staircase diagram, but the ratio of chews to *bites* is expected to be an incidental outcome of other processes, and not a determining factor itself.

#### 3.4.2. Jaw Movement Types and Transitions

Across Experiment 1 in its entirety, there were 239 instances of a chew–bite, compared to 641 pure bites, making chew–bites an important component of the jaw movement mix. The joint probability matrix for all bite masses together shows the dominance of pure chews in the overall mix and of pure-chew to pure-chew pairings (Table 2). The proportion of such pairings responded strongly when separated by bite mass. The transition matrix for all bite masses together shows that, in general, it was rare for a chew–bite to be followed by another chew–bite or a pure bite (Table 2). The separate matrices for each bite mass show that almost all those cases occurred at the lowest bite mass of 0.6 g. Pure bites were rarely followed by chew–bites and only at the lowest bite mass. Consecutive pure bites occurred 119 times in Experiment 1, allocated inversely to bite mass: there were 71, 40, and 8 consecutive pure bites at bite masses of 0.6, 1.2, and 2.4 g, respectively. The fact that animals were capable of consecutive pure bites of 2.4 g each indicates that the maximum bite mass of 2.4 g was not excessive relative to the capacity of the mouth. The proportion of *from*–*to* jaw-movement pairs that were consecutive pure chews increased greatly with the increasing bite mass, from 47% (0.6 g) to 83% (2.4 g).

#### 3.4.3. Simple Linear Regression

**Figure 5:**
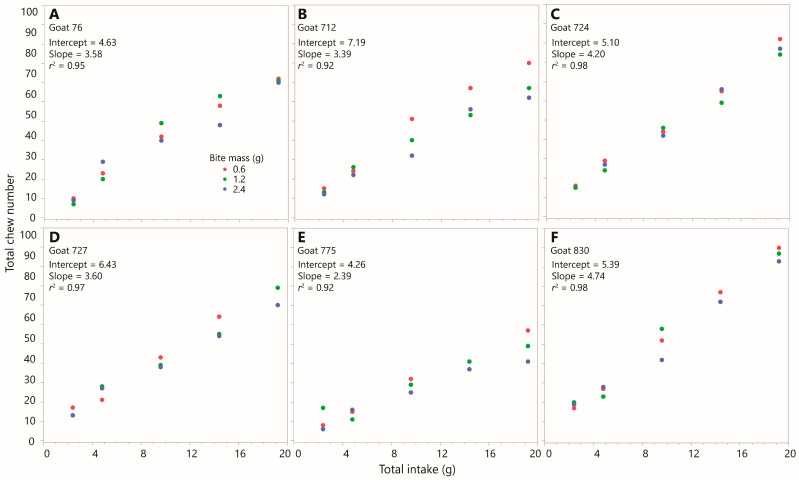
The relationship between chew number (*y*-axis) and intake (*x*-axis) in Experiment 1, by goat and bite mass. Equal scaling is used across all panels. Regression equations shown are for pooled data at the animal level. Linear regression statistics for the relationships at the animal × bite mass level are given in Table 3. (**A**) Goat 76; (**B**) Goat 712; (**C**) Goat 724; (**D**) Goat 727; (**E**) Goat 775; (**F**) Goat 830.

Strong linearity was evident in the within-animal relationship between chewing and intake (Figure 5). The simple linear regression of the total number of chews versus intake, for each combination of goat and bite mass, explained at least 90% of the variation (*r*^2^) across all 18 regressions, and at least 98% of the variation was explained in half of them (Table 3). The linear model was highly significant in all 18 regressions (maximum *p*-value = 0.008). The intercept term was always positive, but its *p*-value was >0.1 in all but four instances. Pooling data across bite masses did not markedly increase the spread of points around the trend line, and the *r*^2^ values were >0.91 across all six animals. There was no clear visual evidence of a consistent effect of bite mass on the relationship. But pooling across animals introduced considerable variation around the trend line, consistent with a strong animal effect on the chewing effort (i.e., slope of the regression line) (Supplementary Figure S4). The slopes for the six goats (pooled across bite masses) ranged from 2.4 to 4.7 chews g^−1^.

#### 3.4.4. Mixed Model Analysis of Experiment 1

In the mixed-model analysis of the chewing coefficient, the bite mass was significant (*p* = 0.0306), and its coefficient was negative. The decline in chewing coefficient over the range of bite masses examined was small: a 400% increase in bite mass was associated with only an 11% decrease in the chewing effort. The log-intake was highly significant (*p* < 0.0001), and the coefficient was also negative. The prediction equation for the chewing coefficient (*y*) was

*y* = 6.52 − 0.230 × Bite mass − 0.806 × ln[Intake]

**Figure 6:**
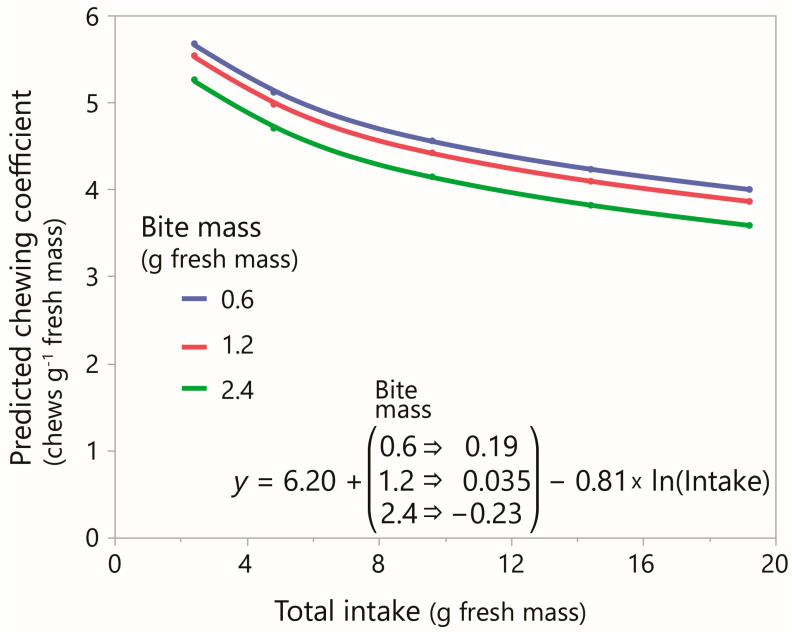
The relationship between predicted chewing coefficient (chews g^−1^ fresh mass; *y*-axis) and total intake (g fresh mass; *x*-axis) for the three nominalized values of bite mass in Experiment 1. In the equation, *y* is the predicted chewing coefficient. The intercept adjustment factor is selected according to the categorical bite mass, and intake is logarithmically transformed.

The chewing coefficient showed a substantial response when considered over the wide range of intakes examined. Whether or not the lower end of the intake range, included to strengthen the regression analysis, would amount to a substantial proportion of daily consumption under most foraging conditions is discussed later (Section 4.2). The logarithmic nature of the response to intake implies that it is highest at the lower end of the intake range, as shown in Figure 6 using nominalized bite mass for illustrative purposes.

#### 3.4.5. Jaw Movement Rhythm of Experiment 1

**Figure 7:**
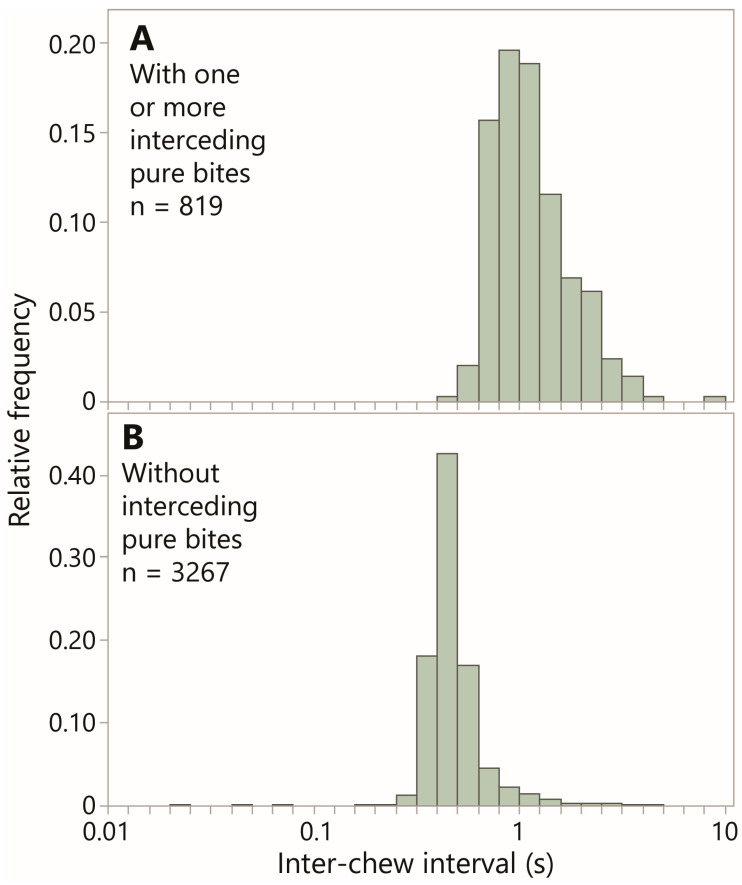
The frequency distribution of inter-chew interval (log scale) in Experiment 1. Panel (**A**) is based on intervals containing one or more interceding pure bites. Panel (**B**) is based on intervals without interceding pure bites. The inter-chew interval was calculated to the previous chew action, which could be the preceding event in the jaw movement stream or be interceded by one or more pure bites, which are ignored in the interval calculation.

The jaw movement rhythm was described in terms of the interval between the nearest chew actions (either or both of which could be a pure chew or a chew–bite); these can be successive events in the jaw movement stream, or they can be interceded by one or more pure bites. The frequency distribution of inter-chew-action interval for these two groups is shown in Figure 7. Even at the fine temporal scale of a few seconds, a logarithmic scale was most suited to the data. For consecutive chew actions, the median interval was 0.45 s (133 events min^−1^); for non-consecutive chew actions, the median was 1.1 s. Given that most non-consecutive chew actions have only a single interceding pure bite, a value approximately double the consecutive-chew interval was expected due to the two duty-cycles of the jaw movement was entailed, even if they were not identical in their vertical and lateral components of motion [30]. But the frequency distributions reveal a large area of overlap between them; consecutive chew actions (the vast majority of which are consecutive pure chews) can be multiples of the median, while the time between two non-consecutive chews can be less than double the “base interval” of 0.45 s. This value appeared to be consistent across bite masses (Supplementary Figure S5).

#### 3.4.6. Rhythmicity and Its Deviations

**Figure 8:**
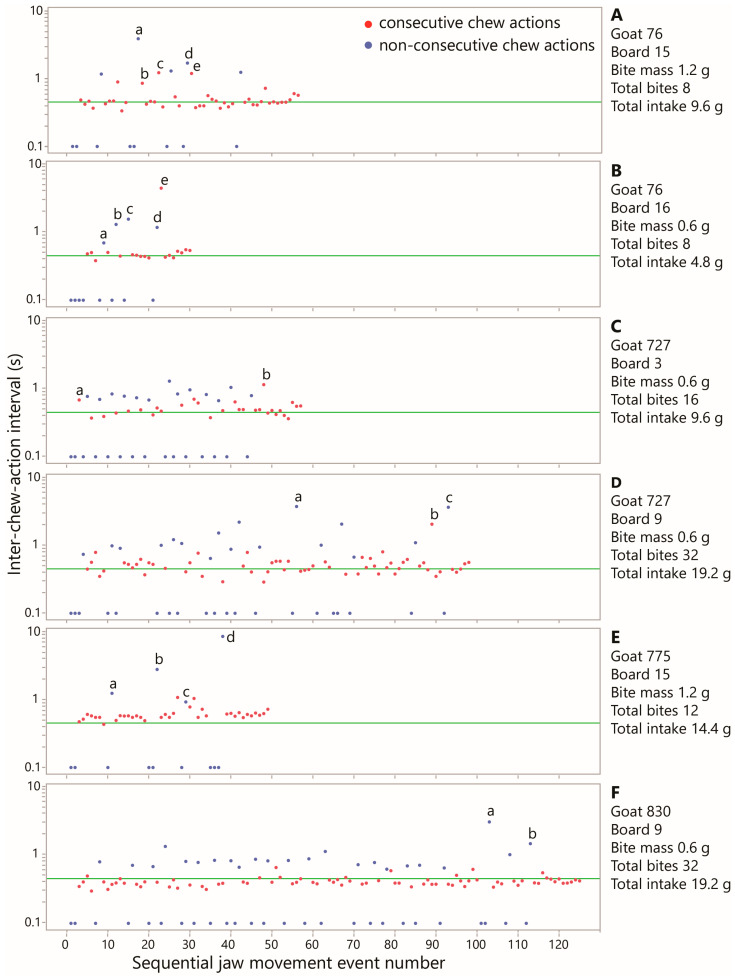
Examples of event-based plots showing the inter-chew-action interval (*y*-axis, log scale) over the course of six sessions of Experiment 1. The position of pure chews in the jaw movement sequence is indicated by an interval of 0.1 s. Color separates consecutive chews versus inter-chew intervals containing one or more pure bites. Lowercase letters denote elevated interval values that were examined in the video and are explained in Appendix A. The horizontal reference line (green) of 0.45 s indicates the approximate global base interval. (**A**) Goat 76, Board 15; (**B**) Goat 76, Board 16; (**C**) Goat 727, Board 3; (**D**) Goat 727, Board 9; (**E**) Goat 775, Board 15; (**F**) Goat 830, Board 9.

The dominance of the above “base interval” was evident in the event-based plots of each intake session for each individual animal (examples are shown in Figure 8). Nevertheless, the event-based plots deviated significantly from metronome-like regularity. That would have manifested as two narrow bands of points, one in the region of 0.45 s, comprising consecutive chews only, and a second, sparser band of points in the region of 1 s for chews with interceding pure bites (mostly one). The closest examples to that are panels C and F of Figure 8, but they were not the rule. The ubiquitous irregularities are not measurement errors but reflections of the nuances and subtleties of a biological process. This was illustrated by selecting the more extreme intervals in the event-based plots (labeled with lower-case letters in Figure 8) and examining the corresponding video to understand their cause. The main reasons for elevated intervals were the significant use of manipulative upper-lip movements in bite formation; manipulative non-sound-producing jaw movements to organize material in the mouth; multiple intervening pure bites; momentary vigilance freezes or startles that pause jaw activity; and distractions of a few seconds without jaw activity. (A detailed point-by-point explanation is given in Appendix A.) These behaviors reduce the degree of rhythmicity in jaw activity but are integral to active foraging by goats even in a highly controlled and simplified foraging arena.

### 3.5. Satiety-Level Experiment

#### 3.5.1. Data Overview of Experiment 2

The total amount of vegetation consumed in the entire experiment was 777.6 g, and 3056 chew actions were performed in consuming it. This yields a coefficient of 3.9 chews g^−1^ intake, slightly less than the value obtained in Experiment 1. A total of 324 bite actions were performed, yielding a ratio of 9.4 chews bite^−1^, very different to the value of 4.3 obtained in Experiment 1. Because there was a single bite mass in Experiment 2, it is not possible to differentiate between the abilities of intake and bite number to predict the chewing requirements. The relationship between chews performed and intake for the pooled test sessions of Experiment 2 is shown in Supplementary Figure S6; the relationships for each animal are shown in Supplementary Figure S7. The total number of chew actions performed by an animal in order to consume a total of 130 g of foliage across all treatments ranged from 331 to 593. The total net ingestion time (based on the first and last chews) was 1937 s, yielding an overall instantaneous intake rate of 24.1 g min^−1^ (≈9.6 g DM min^−1^), highly consistent with the value of 24.8 g min^−1^ obtained in Experiment 1.

#### 3.5.2. Jaw Movement Rhythm of Experiment 2

**Figure 9:**
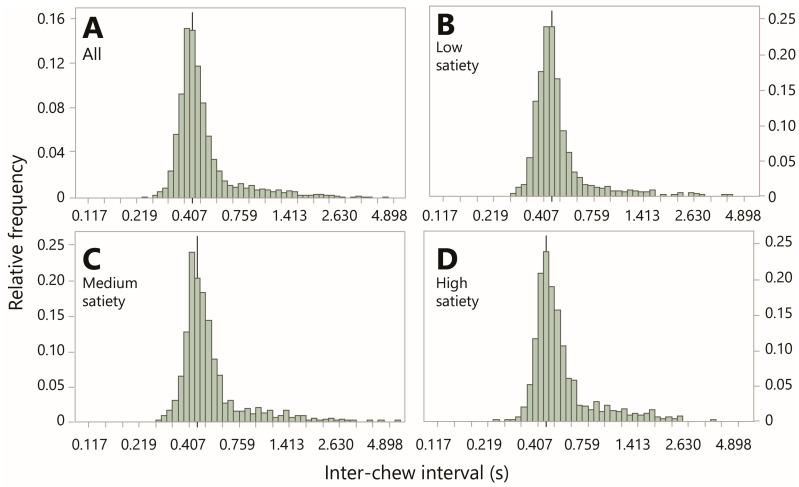
The frequency distribution of inter-chew interval (log scale), for Experiment 2. The inter-chew interval was calculated to the previous chew action, which could be the preceding event in the jaw movement stream or be interceded by one or more pure bites, which are ignored in the interval calculation. Intervals in the region of the distribution’s peak are expected to be derived from consecutive chew actions. Panel (**A**) shows pooled results for the entire experiment. Panel (**B**) shows results for low satiety. Panel (**C**) shows results for medium satiety. Panel (**D**) shows results for high satiety. The vertical reference lines are at 0.45 s.

The global frequency distribution of the inter-chew-action interval is shown in Figure 9A. The median interval was 0.46 s (130 events min^−1^). In the analysis of the rate of jaw movement, intake was not significant (*p* > 0.8), but the satiety level was highly significant (*p* < 0.0001); the rate of jaw movement increased from 104 min^−1^ at high satiety to 118 min^−1^ at low satiety level. Despite the difference in these overall rates, no shortening of the fundamental “base interval” of 0.45 s was apparent in the frequency distributions of inter-chew interval (Figure 9B–D). Rather, it was the consequence of a larger proportion of jaw movement transitions being consecutive pure chews (which have the shortest interval), in turn caused by an elevated chewing coefficient. This is consistent with the inter-chew-interval for non-consecutive chew actions (1.1 s) being *more* than double the base value.

#### 3.5.3. Mixed Model Analysis of Experiment 2

In the analysis of the chewing coefficient, the intake was not significant (*p* = 0.1561), while the satiety level was significant (*p* = 0.0252). The least square means declined with increasing satiety (4.3, 3.9, and 3.7 chews g^−1^ for low, medium, and high satiety, respectively). The effect was not large; a change from low to high satiety level is associated with a 14% reduction in chewing effort.

### 3.6. Intake Prediction

**Figure 10:**
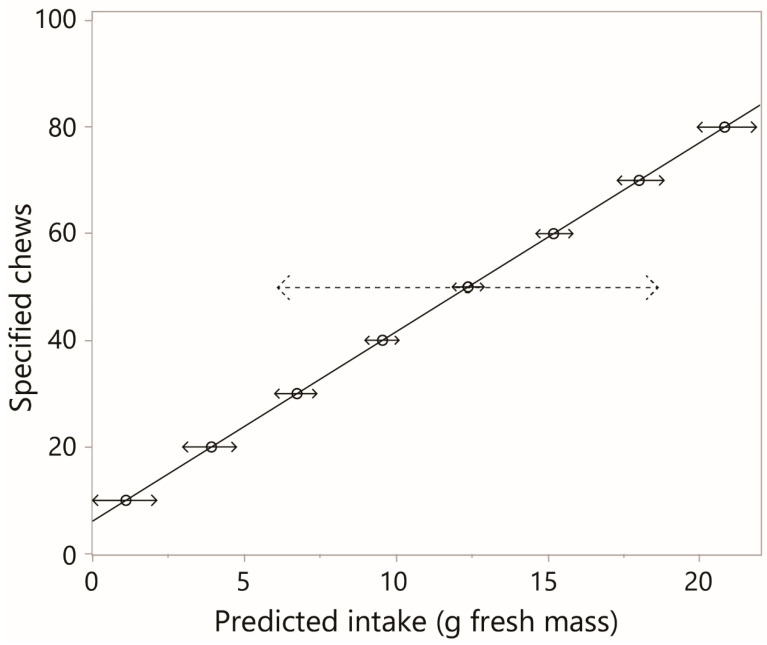
Inverse regression for the purposes of predicting intake from chews, based on simple linear regression of chews versus intake for the pooled data of both experiments. Computed over a range of 10–80 chews, in intervals of 10 chews. Solid arrows indicate ±95% confidence interval with respect to an expected response. Dashed arrows are the equivalent with respect to an individual response.

In the hypothetical scenario of obtaining chew counts from “similar” goats foraging carob monocultures, with no further information, the most elementary approach to intake prediction would be to first pool the data from both experiments, given they are indistinguishable when pooled (Supplementary Figure S8), and perform linear regression of chews (*y*) versus intake (*x*). Using inverse regression, intakes and their 95% confidence limits were computed over a range of chew counts (Figure 10). For counts of at least 50, the confidence limits span less than 10% of the estimated intake. This ratio climbs as the chew count declines. These confidence limits are with respect to an expected response at the population level; as would be expected, they expand substantially when computed with respect to an individual response, as shown for a single specified chew count in Figure 10. As will be discussed (in Section 4.9), substantial improvements are expected when predictions are more individualized.

## 4. Discussion

### 4.1. Indicators of Stability

**Figure 11:**
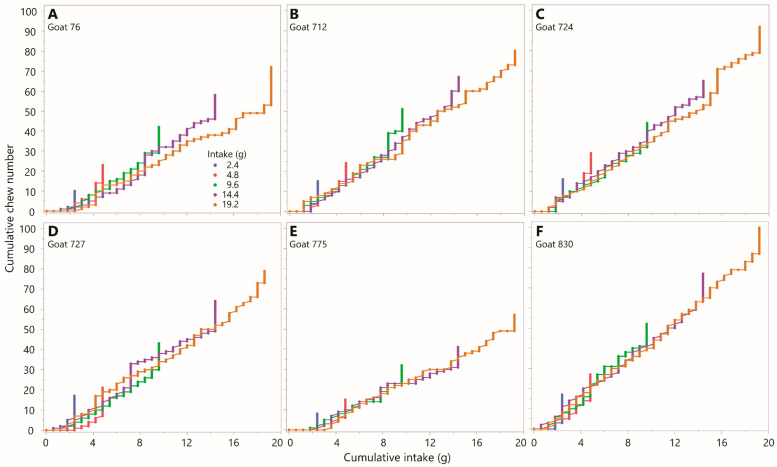
Chew versus intake staircase diagrams from Experiment 1 showing the chronological sequence of chew actions (*y*-axis) and bite actions converted to intake (*x*-axis), expressed cumulatively from lower-left to upper-right, for five levels of total intake based on a single bite mass of 0.6 g throughout. Each panel corresponds to one experimental animal. Equal scaling is used across all panels. (**A**) Goat 76; (**B**) Goat 712; (**C**) Goat 724; (**D**) Goat 727; (**E**) Goat 775; (**F**) Goat 830.

Although there is a strong biomechanical component to jaw movements [31,32], the chewing coefficient is nevertheless not a physical constant but an outcome of the behavior of a complex organism upon which myriad factors impinge constantly. These could be oronasal senses, visual cues from the surroundings, or, for that matter, picking leaves from a plywood board in the absence of conspecifics. It is really the *variability* of the chewing effort that is important. As the period of examination shortens, the stability of the chewing effort is expected to decline due to short-term random variation. The present study operated at a time scale one or two orders of magnitude less than estimates of intake would seek to operate (most commonly, one day). It was therefore encouraging that there was reasonable similarity between the experiment-wise chewing coefficients of Experiments 1 and 2 for each animal (linear regression *p* = 0.007). This suggests that the large variation in chewing effectiveness obtained across animals within each experiment are animal effects and not random variation around a shared chewing effectiveness for large time scales. The usually-high degree of overlap between the within-animal trajectories on the chew versus intake staircase diagram for different intakes at constant bite mass (Figure 11) is evidence of reasonable stability even at short time scales.

### 4.2. The Importance of Bite Mass

Under natural conditions, the mass of the bites removed by goats while foraging on shrubs and trees would be expected to vary greatly over short periods of time, even within the same plant species. Hence, a given intake can be accumulated via numerous combinations of bite number and bite mass. Experiment 1 tested whether the chewing effort is influenced by bite mass and found that it does not appear to be modulated by bite mass in a substantial way: a fourfold increase in bite mass (from 0.6 to 2.4 g) reduced the chewing coefficient from 4.7 to 4.3 chews g^−1^, a small improvement (reduction) of only 9%. The ability to detect significant differences at this resolution was facilitated by the control and precision afforded by the entire methodology, including, not least, acoustic monitoring. Similarly, the absolute effect of the bite mass is fairly small, which is an encouraging result in terms of the ability to estimate intake amounts from chew counts.

One caveat regarding the chewing coefficient that emerged from Experiment 1 was the finding that the intake level itself had a role to play; as intake declined from 19.2 to 2.4 g there was an overall increase in chewing coefficient of 43%. The simplest explanation for this increase is that chews were predominantly being performed when herbage loading in the mouth was below capacity, be it during the initial process of filling the mouth or the terminal process of emptying it. The intake level of the treatment may not have reached mouth capacity, forcing an inefficiency. This raises two questions, which are discussed below: How important is this in “the real world”? What, if anything, should be done about it?

### 4.3. Importance of Mouth Filling and Emptying

What makes the low-intake-level treatments of Experiment 1 of interest is that the filling and emptying phases formed a substantial, if not total, proportion of all jaw movements. It could be argued that cattle, sheep, and goats do not present such a pattern of behavior when engaged in active grazing bouts on abundant herbaceous pastures. Although there may be interruptions in jaw activity that are flanked at both ends by an emptying or filling phase, in aggregate these are unlikely to impact the supposed “steady-state” chewing coefficient. However, as forage conditions worsen, and the searching time between successive bites is inserted into the timeline of jaw activity, the prevalence of emptying and filling phases must grow. That is assumed to impact the chewing coefficient: each chew action performed when mouth fill is low generates less-than-potential “product”. We would, therefore, like to know the proportion of all chew actions that is associated with emptying/filling phases, regardless of the length of the intervening bouts of jaw movements.

As a first approximation, an analysis was conducted of the acoustically-derived timelines of jaw activity reported in Ungar and Horn [7], obtained under extensive grazing conditions. A time threshold, *t*, was set for the inter-jaw movement interval above which it is assumed, retrospectively, that the last *m* jaw movements preceding the interval were chews performed under “emptying” conditions of reduced mouth fill, and the first *n* jaw movements of the succeeding sequence (presumed to include bites and chews) were performed under “filling” conditions of reduced mouth fill. Implicitly, all other jaw movements occur at a higher level of mouth fill that might be regarded as “steady-state” or “normative”. A simple measure of the importance of empty-and-fill phases is to calculate the proportion of all jaw movements devoted to them.

As the parameters *t*, *m,* and *n* are unknown, different values were explored, as summarized in Supplementary Figure S9. For the shortest thresholds in the region of 3–4 s, approximately one quarter of the total events can be attributed to mouth emptying or filling phases. The proportion declines with an increasing threshold, more steeply at first, and plateaus in the region of 10%. The seriousness of the issue depends on the assumed values for *t*, *m*, and *n*, for which empirical data are currently lacking. If the problem is deemed serious, what could or should be done about it depends on how the notion of chewing coefficient is interpreted.

### 4.4. Classic Interpretation of Chewing Coefficient

The classic interpretation views the mass ingested as the driving variable that governs chewing. However, when the mouth fill is below its normative potential for whatever reason, the absolute amount of herbage processed by a chew is reduced, necessitating, locally, more chews per unit mass. To account for this, it might be possible to empirically estimate separate chewing coefficients for low and high intake levels and use Supplementary Figure S9 as a guide to weighting them in a single chewing coefficient. Extracting the number of jaw movements in a bout is straightforward given the timeline of jaw movements generated by acoustic sensing.

### 4.5. Emergent Interpretation of Chewing Coefficient

There is an alternative interpretation of the chewing coefficient, developed fully in Appendix B, whereby no “special treatment” is needed for phases of low mouth fill. In this view, instead of being mass-based, chewing is governed by a somewhat different rule: the material contained in a bite must be subjected to a constant number of chew actions (denoted *V*) in order to make the material “swallow-ready”. Crucially, a single chew advances all cohorts of bites that happen to be in the mouth, by one step in this process, irrespective of how much material is in the mouth. Thus, the chewing coefficient is an emergent property of this process and is not expected to be a universal constant. The value of *V* selected was based on minimizing the sum of excess and deficit chews predicted by applying the *V*-rule to the observed data. When determined separately for each animal, the optimum value (V*) ranged from 10 chew actions (for goat 775, which had the lowest chewing coefficient) to 19 chew actions (for goat 830, which had the highest chewing coefficient). The absolute error numbers were low, and a strong linear relationship was obtained between the predicted and observed chew number (*p* < 0.0001; *r*^2^ = 0.68). The model did not generate implausible levels of mouth fill. The global pooled frequency distribution of the mouth fill of Experiment 1 indicated that 90% of values did not exceed 4.8 g, and the median mouth fill was 3 g: both eminently reasonable values. Although a few examples could be found, there was no compelling evidence that animals maintained a steady-state of relatively high mouth fill. Even under the ideal conditions furnished by the methodology, filling and emptying of the mouth (or, at least, large fluctuations in mouth fill) did not just occur at the extremities of an ingestive bout, but more widely.

It follows that the variable of interest that is expected to modulate the chewing coefficient is the average mouth fill across all chew actions of an ingestive bout. There did appear to be an inverse relationship between the observed chewing efficiency in Experiment 1 and the simulated mean mouth fill. It is shown algebraically in Appendix B that the predicted chewing coefficient is equal to the ratio of V* to mean mouth fill. This yielded a strong relationship between the predicted and observed chewing coefficient.

Using this approach, the issues of filling and emptying phases, or low-intake-level situations, are essentially bypassed. The chewing coefficient, which is used to convert chew counts to intake amounts, is derived (or emerges) from a different underlying principle to that of dividing chew number by mass ingested.

### 4.6. Reexamination of the Bite Mass Effect

How might the small reduction in chewing coefficient with increasing bite mass be interpreted under the *V*-based model? The discrete jumps in mouth fill that accompany the two higher bite masses make it difficult to be definitive, but it appears that the mean mouth fill increased with the bite mass (3.0, 3.3, and 4.0 g mouth fill for bite masses 0.6, 1.2, and 2.4 g). If all other assumptions remain in effect, this should lower the chewing coefficient via the ratio of V* to the mean mouth fill.

### 4.7. The Importance of Satiety Level

We emphasize that the satiety-level treatments should not be seen as representing precise physiological states associated with a defined level of glucose in the blood. Such determinations were beyond our methodological and logistic capacity. Our goal was to investigate the robustness of the chew–intake relationship to different levels of satiety as practically experienced by a browsing herd. The three satiety-level treatments were chosen to reasonably represent the early, mid, and terminal phases of a grazing session under natural conditions.

We found that satiety does indeed modulate the chewing effort, but only to a small extent and not in the direction expected: hungry animals invested 15% *more* chews per unit intake than did sated animals. We can only speculate as to why that might be. Additional chewing may facilitate the chemical activity of saliva in the mouth and the breakdown of digesta in the rumen [33], so as to alter rumen chemistry and hasten hunger relief. Alternatively, if some feedback existed whereby low satiety elevates the rate of jaw movement, it might reduce the effectiveness of each chew, requiring more chews, thereby elevating the chewing effort. But the unchanging “base interval” of 0.45 s across satiety levels (Figure 9) leans strongly against this. In terms of the overall time budget of intake, the data suggest that the effects of the elevated rate of jaw movement and elevated chewing effort cancel each other out.

The modest effect that satiety was found to have on the chewing effort is, perhaps, a complication, but hardly an impediment to the future prediction of intake from chews. For some purposes it could be ignored. Where it is preferred not to ignore it, satiety can be readily gauged from the same timeline of ingestive jaw movements that is generated by the acoustic sensor in order to count chews.

### 4.8. Bite Masses in Broader Context

How do the pre-determined bite masses employed in the present study compare to those encountered in the field? A study was conducted at Ramat Hanadiv Nature Park in the same area of Mediterranean garrigue vegetation that was exploited by the goat herd of the present study [29]. Using a technique that mimicked the bites of a focal animal, bite-sized units of vegetation were clipped and collected. The samples encompassed 23 non-herbaceous species, including 14 woody species (carob among them), and various size categories within each species. The population of bite mass estimates ranged from 0.01 g DM (*Rhamnus lycioides*) to >5 g DM (*Pistacia lentiscus*). Our low-bite-mass treatment of ≈0.25 g DM was just below the median of this population, and our high-bite-mass treatment of ≈1 g DM was at the 80th percentile. If, as a first approximation, animals maintain a chewing effectiveness of ≈10 chews g^−1^ DM intake across bite masses, then values that are significantly lower than 0.1 g DM would require runs of consecutive pure bites to maintain fill level in the conceptual tank, and bite actions would dominate the jaw movement sequence. Although a trend in that direction with declining bite mass was seen in the runs analysis (see Appendix B, Table A1), lower bite masses would be needed to confirm that.

### 4.9. From Chews to Intake

The question of how well chew counts can predict intake amounts can be addressed in different ways. Within the narrow world of carob monoculture, the most rudimentary application of the results obtained would be prediction at the population level, with no further information on the target animals. Obviously, the less similar they are to the test animals of this study, the less reliable the predictions are expected to be. The inverse regression presented in Section 3.6 showed that the potential exists for usefully precise predictions if the sampling size is large. Predictions for an individual animal would not be reliable. To obtain predictions better tailored to the individual animal, the prediction equation can incorporate the most basic animal features expected to impact the chewing coefficient, such as the live weight and incisor arcade width [24], which would also need to be determined for the target animals (there were too few animals and insufficient variation along the two axes to apply this approach here). The ideal level in prediction is the local determination of the chewing coefficient, which might be feasible if working with compliant animals. At this level, the inverse regression is not relevant anymore, but the expectation is to obtain highly accurate estimates of intake from chew counts. Most importantly, the results of the present study create a null expectation that if a chew–intake calibration were to be conducted with a strongly contrasting breed of goat and vegetation, a tight linear chew–intake relationship (within-animal) would nevertheless be obtained, and the chewing coefficient would be fairly stable. That could simplify the effort needed to be invested in the determination.

The potential utility of counting chews to predict intake when woody vegetation is being browsed lies in the variability of the chewing effort among plant species. It is difficult to find points of comparison in the published literature that are not based on stall-fed animals (or hand-fed animals in the case of Wang et al. [15]). Of the 90 data sources in the meta-analysis of Boval and Sauvant [34] that dealt specifically with cattle, sheep, or goats *at grazing*, only four used goats, of which none monitored chewing. Estimation of the chewing coefficient based on acoustic sensing and hand-constructed patches could be adapted for a wide range of woody species browsed by goats in a given forage environment. However, this opens up new challenges of deciphering which species are being consumed or at least their proportions in the total diet.

Certain habitats offer grazing animals a combination of woody and herbaceous vegetation, introducing an additional layer of complexity that must be examined. This is necessary, because the variability of the chewing coefficient among these very different dietary components is unknown and may be substantial. One possible solution is to combine acoustic monitoring with the placement of a video camera on the goat’s horn and to apply image-processing techniques to distinguish between woody and herbaceous vegetation and identify the woody plant species. Preliminary trials conducted by the authors demonstrated that this approach makes it possible to manually identify, at any given moment, the food source being consumed by the studied animal.

In the experiments reported here, we directly addressed the two factors identified as having the highest potential to be problematic and found that they do not constitute a limiting factor for the conversion of chew counts to intake amounts. Moreover, there does appear to be a general tendency to maintain chewing efficiency as constant as possible, including a relatively stable chewing coefficient. However, it is important to clearly emphasize that additional factors not examined here may significantly affect the chewing coefficient, such as seasonal effects, physiological states, and other unforeseen influences that emerge over larger timescales. These issues will need to be addressed in future studies.

Finally, we note that in real life, pure bites would be detected by acoustic sensing, even though they were intentionally bypassed in this study. They would need to be identified as such and excluded from chew counts. Failing that, the overall proportion of pure bites in the jaw movement stream could be corrected for using normative values derived from sequenced datasets. On woody vegetation, at least, bite sounds are expected to be very different in quality to chew sounds, which should ease classification. But this is nevertheless a complication that acoustic sensing technology would need to contend with. However, if it transpires that bite actions elicit distinctive acoustic signatures for different browse species, then the door is open to exploiting species-specific coefficients.

## 5. Conclusions

Acoustic sensing enabled an in-depth study of the relationship between chewing and intake in goats consuming woody vegetation foliage. The animals displayed considerable consistency in the way they sequenced chew and bite actions in the course of depleting the herbage offered. The overwhelmingly primary determinant of the chewing effort was the amount consumed. The chew-to-bite ratio was incidental. The effects of the bite mass on the chewing effort were relatively small, as were the effects of the level of satiety. A case can be made that the chewing coefficient emerges from a more basic rule governing the performance of chew actions. This has implications for how chew counts might be converted to intake amounts more widely.

Despite their deficiencies, hand-constructed swards of one kind or another furnish highly accurate determinations of woody vegetation intake that can be paired with acoustically determined chew counts. The method could be used to widen the range of species studied at this level. An important future test of this sensor technology is to examine the stability of the chewing effort across a wide variety of woody plant species. This could be achieved without hand-constructed swards, using simpler test arenas, such as before-and-after weighing of entire branches or bushes to estimate the intake. The prospect of being able to estimate the intake from acoustically determined chew counts would seem to strengthen the case for the continued development of this sensor technology.

## Figures and Tables

**Table 1:** The design of Experiment 1 to determine the response of chewing effort to different bite masses over a range of total intake levels. The number of bites removed was controlled by populating an equal number of positions in a baseboard with a bite-size unit of vegetation of the specified mass. The fifteen treatment combinations were tested on six goats.

Total Intake (g Fresh [Dry] Mass)	Bite Mass (g Fresh [Dry] Mass)		
	0.6 [0.25]	1.2 [0.5]	2.4 [1]
	*n* bites removed		
2.4 [1]	4	2	1
4.8 [2]	8	4	2
9.6 [4]	16	8	4
14.4 [6]	24	12	6
19.2 [8]	32	16	8

**Table 2:** The joint probability matrices and transition matrices for consecutive jaw movements, each being a pure bite, a pure chew, or a chew–bite, for a global analysis of Experiment 1 and for each bite mass separately. There were 4341 *from*–*to* pairs in the global analysis.

			Joint Probability Matrices			Transition Matrices		
Bite	*From* jaw		*To* jaw movement			*To* jaw movement		
mass (g)	movement	*n*	Chew–bite	Pure bite	Pure chew	Chew–bite	Pure bite	Pure chew
				%			%	
All	Chew–bite	239	0.2	0.2	5.1	3.8	3.3	92.9
	Pure bite	641	0.2	2.7	11.9	1.1	18.6	80.3
	Pure chew	3461	5.1	9.8	64.8	6.4	12.3	81.3
								
0.6	Chew–bite	148	0.5	0.4	8.0	6.1	4.1	89.9
	Pure bite	355	0.4	4.3	16.7	2.0	20.0	78.0
	Pure chew	1160	7.9	14.9	46.9	11.4	21.4	67.2
								
1.2	Chew–bite	57	0	0.1	3.8	0.0	3.5	96.5
	Pure bite	195	0	2.8	10.8	0.0	20.5	79.5
	Pure chew	1181	4.0	8.6	69.9	4.8	10.4	84.8
								
2.4	Chew–bite	34	0	0	2.7	0.0	0.0	100.0
	Pure bite	91	0	0.6	6.7	0.0	8.8	91.2
	Pure chew	1120	2.7	4.3	82.9	3.0	4.8	92.1

**Table 3:** Linear regression statistics for the relationship between chew number and intake in Experiment 1, examined for each combination of goat (*n* = 6) and bite mass (*n* = 3).

Goat	Bite Mass	Intercept	*p* of Intercept	Slope	*p* of Slope	*r* ^2^
76	0.6	4.25	0.18	3.65	0.0004	0.987
	1.2	2.69	0.70	3.90	0.0055	0.927
	2.4	6.96	0.27	3.20	0.0054	0.928
712	0.6	7.24	0.15	3.98	0.0011	0.974
	1.2	8.66	0.03	3.09	0.0005	0.986
	2.4	5.66	0.23	3.09	0.0023	0.959
724	0.6	5.30	0.17	4.35	0.0004	0.987
	1.2	5.21	0.12	4.01	0.0003	0.989
	2.4	4.50	0.33	4.25	0.0039	0.988
727	0.6	5.59	0.10	3.89	0.0003	0.990
	1.2	5.90	0.19	3.66	0.0011	0.975
	2.4	7.80	0.04	3.23	0.0005	0.986
775	0.6	1.74	0.39	2.86	0.0003	0.990
	1.2	6.91	0.20	2.23	0.0081	0.906
	2.4	4.13	0.24	2.07	0.0032	0.949
830	0.6	4.10	0.02	5.01	<0.0001	0.999
	1.2	6.37	0.26	4.73	0.0012	0.973
	2.4	5.70	0.23	4.47	0.0009	0.980

**Figure A1:**
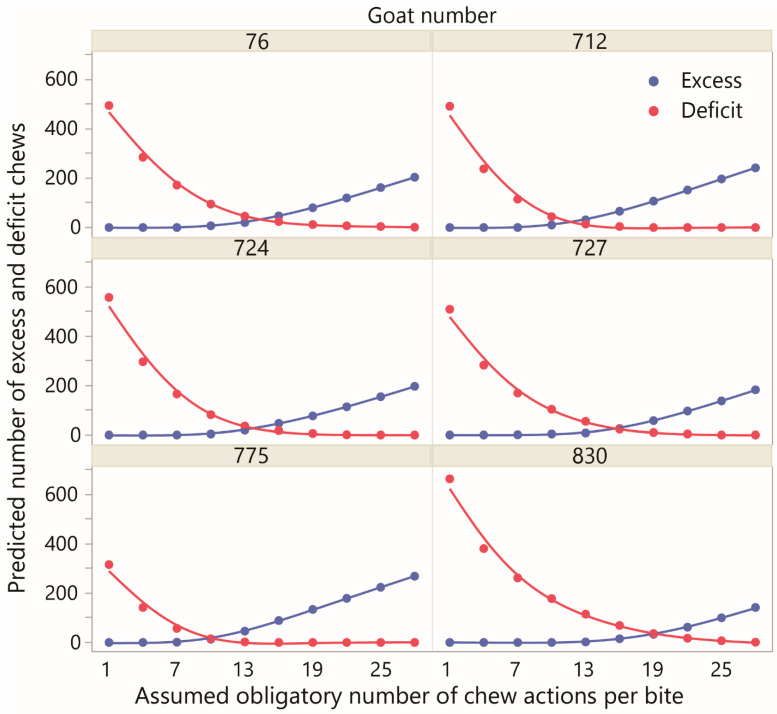
The response of the number of excess predicted chews (blue) and the number of deficient predicted chews (red) (*y*-axis) to the magnitude of the *V* parameter (*x*-axis) in Experiment 1, where *V* is the obligatory chew exposure of a packet of intake (bite). The sum of the two errors is at a minimum (V*) where the lines cross.

**Figure A2:**
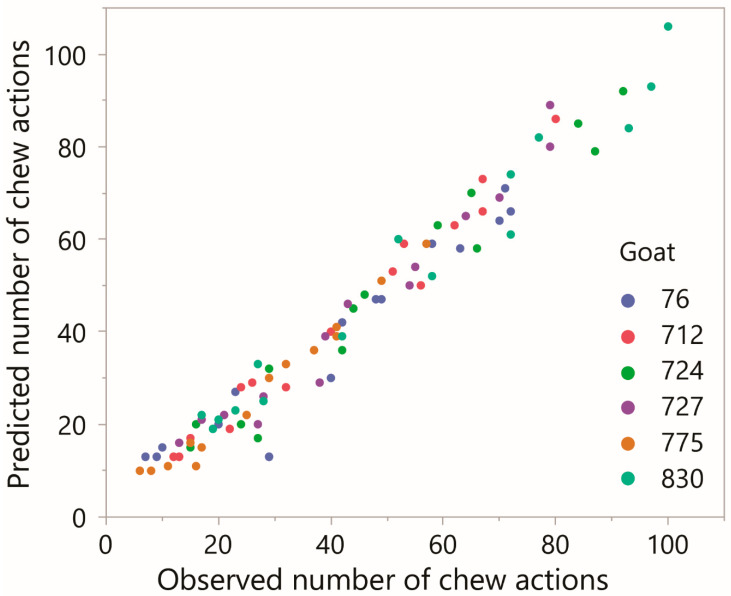
The relationship between the total chew number as simulated by the *V*-based model (*y*-axis) and the observed total chew number (*x*-axis) in Experiment 1 (*n* = 89). The six experimental animals are distinguished by color.

**Figure A3:**
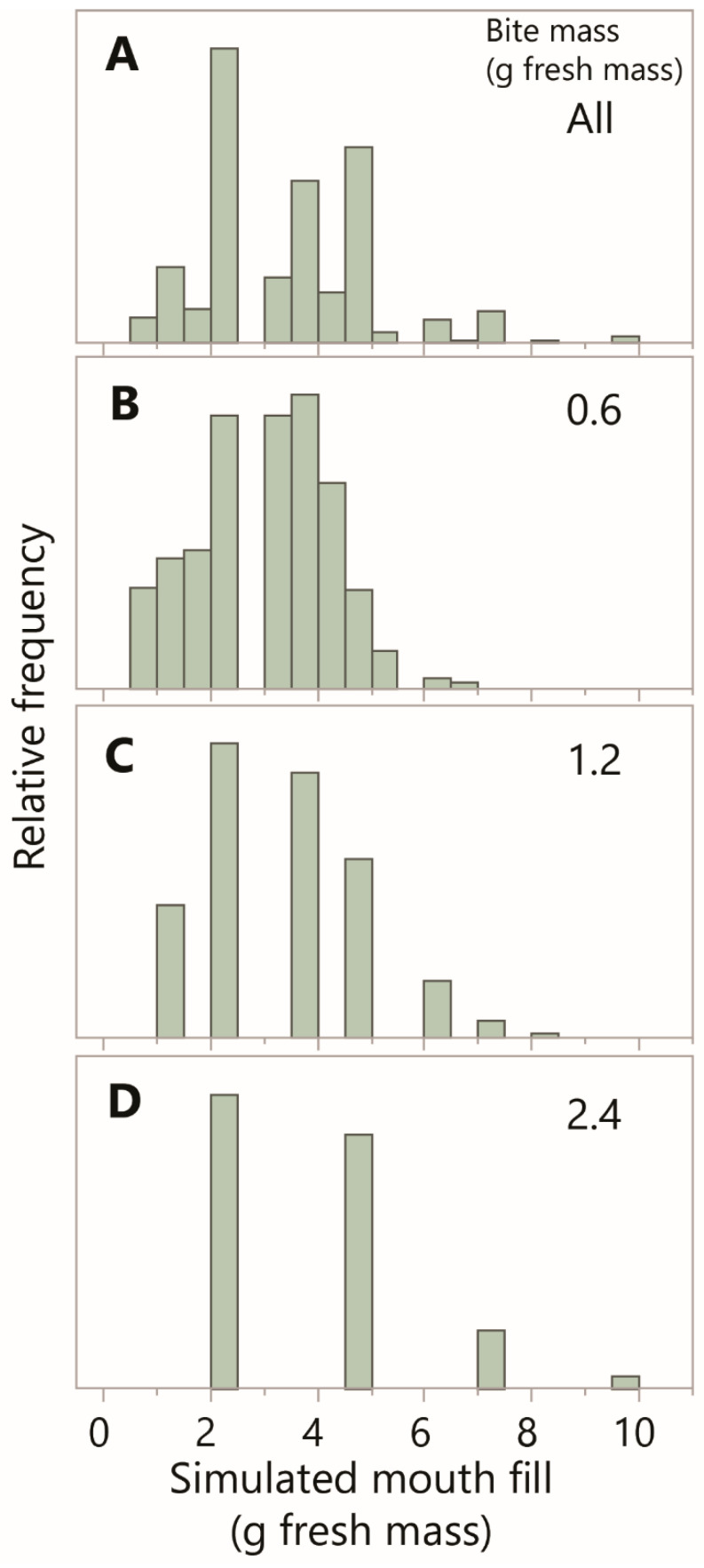
The frequency distribution of mouth fill in Experiment 1 as simulated by the *V*-based model, with *V* individualized by animal, for (**A**) global pooled data; and bite masses of: (**B**) 0.6 g; (**C**) 1.2 g; (**D**) 2.4 g, fresh mass, where *V* is the obligatory chew exposure of a packet of intake (bite).

**Figure A4:**
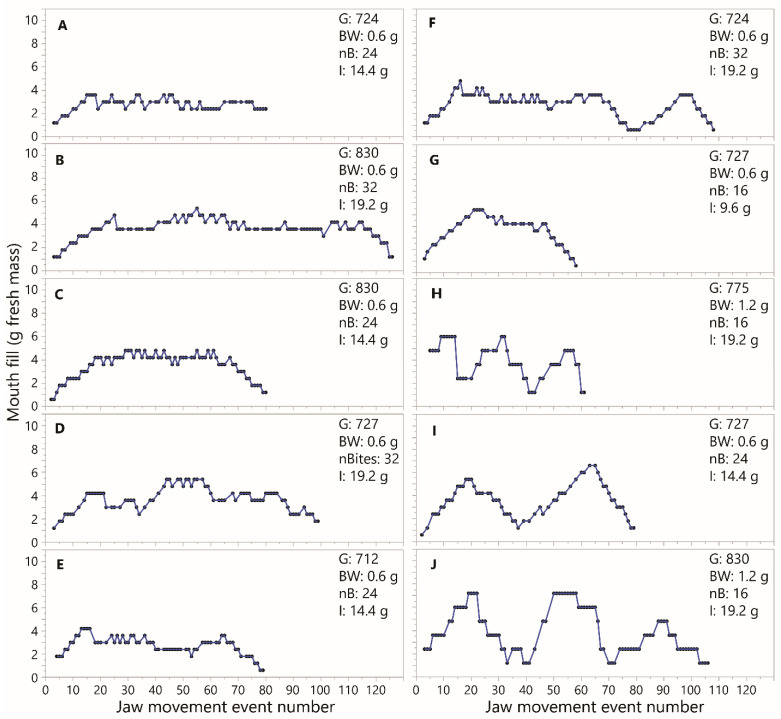
Examples of the simulated mouth fill (*y*-axis) over the course of the depletion of a single hand-constructed patch in Experiment 1, arranged sequentially by chew event number (*x*-axis), according to the model, where *V* is the obligatory chew exposure of a packet of intake (bite). Abbreviations: G: Goat; BW: bite mass; nB: number of bites; I: intake. (**A**) Goat 724; 24 bites; (**B**) Goat 830; 32 bites; (**C**) Goat 830; 24 bites; (**D**) Goat 727; 32 bites; (**E**) Goat 712; 24 bites; (**F**) Goat 724; 32 bites; (**G**) Goat 727; 16 bites; (**H**) Goat 775; 16 bites; (**I**) Goat 727; 24 bites; (**J**) Goat 830; 16 bites.

**Figure A5:**
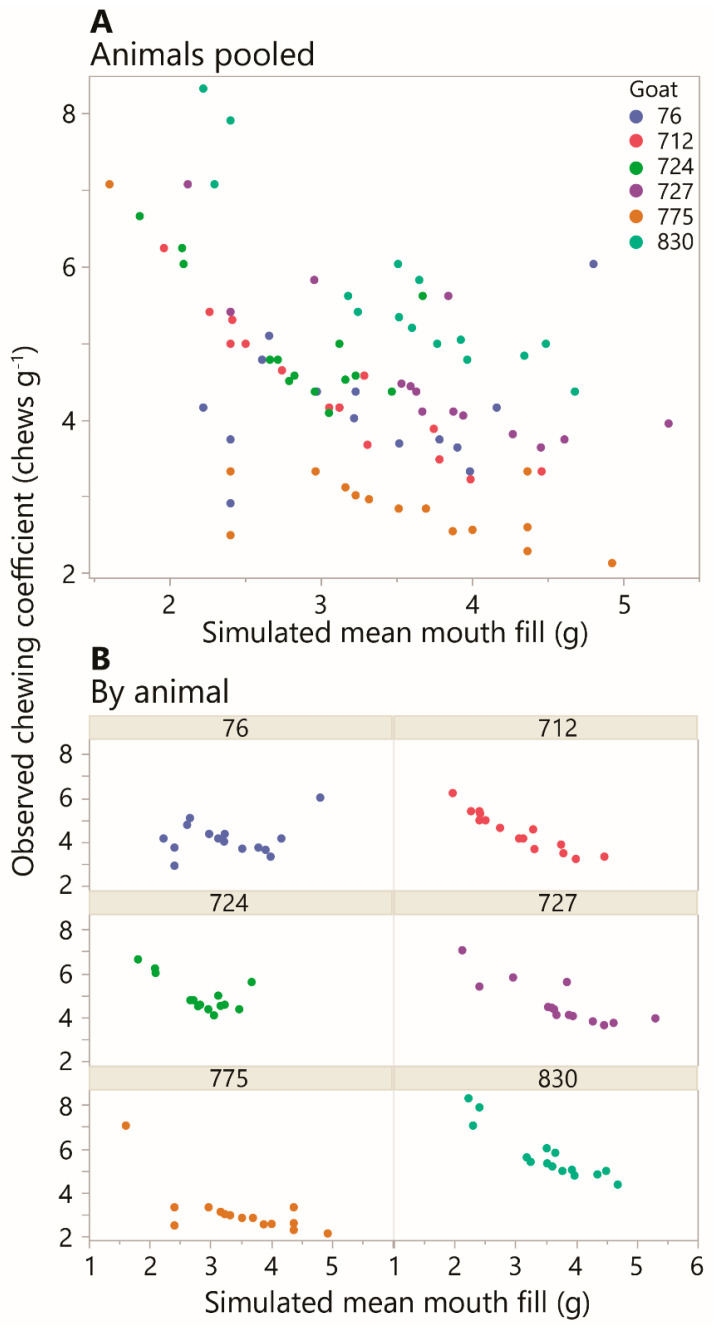
The relationship between the observed chewing coefficient (*y*-axis) and the simulated mean mouth fill (g; *x*-axis), (**A**) overall; (**B**) by animal. The six experimental animals are distinguished by color. Each point represents one foraging bout from a hand-constructed array of carob leaflets (*n* = 89).

**Figure A6:**
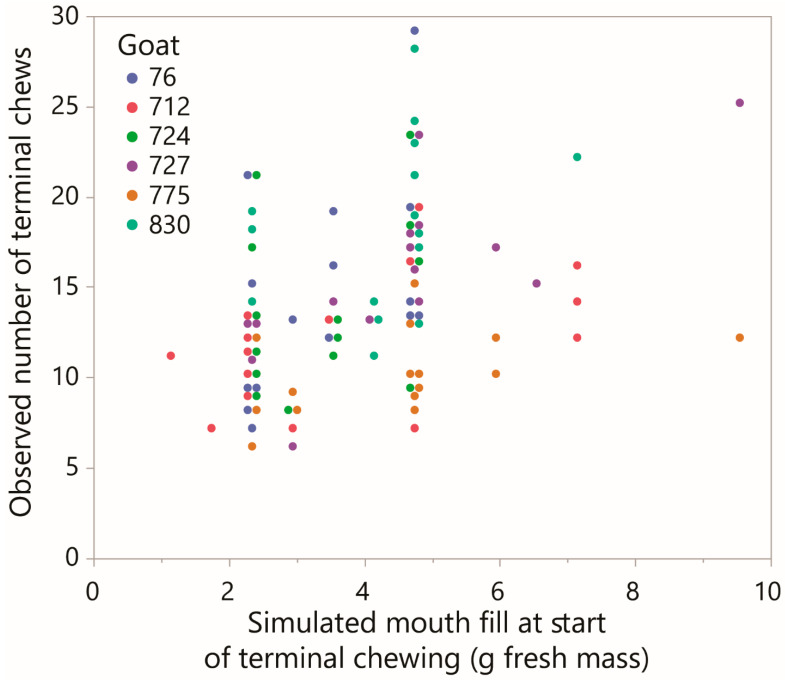
The relationship (or lack thereof) between the number of terminal chews performed (*y*-axis) and the simulated mouth fill at last bite (*x*-axis) in Experiment 1. Each point represents one foraging bout from a hand-constructed array of carob leaflets. The six experimental animals are distinguished by color. Points are jittered to reduce overlap.

**Figure A7:**
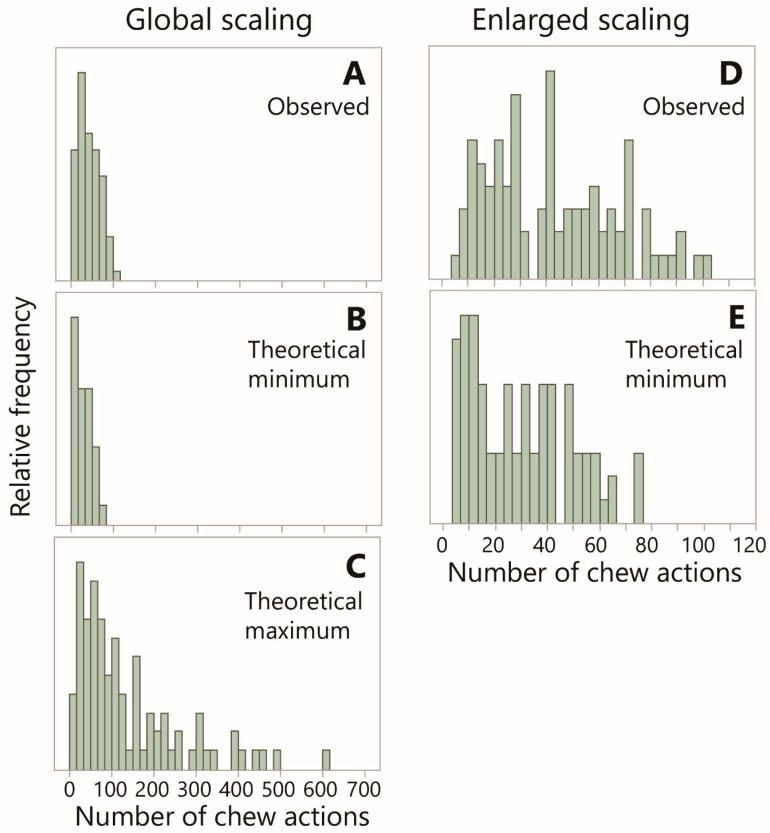
The frequency distributions of observed chewing effort versus theoretical extremes according to the *V*-based model, where *V* is the obligatory chew exposure of a packet of intake (bite). (**A**–**C**) C_obs_, C_min_ and C_max_ on the same scaling. (**D**,**E**) Enlarged scale to compare C_obs_ and C_min_.

**Figure A8:**
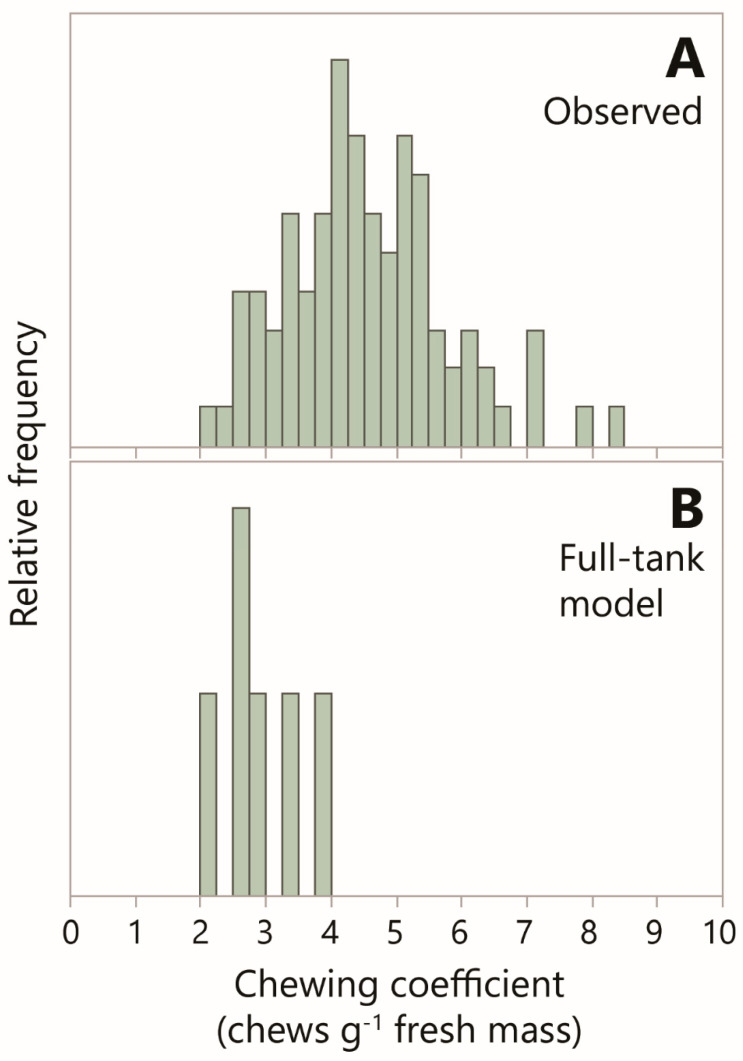
Frequency distributions of the observed chewing coefficient and the chewing coefficient that emerges from application of the *V*-based model operating in full tank mode, where *V* is the obligatory chew exposure of a packet of intake (bite).

**Figure A9:**
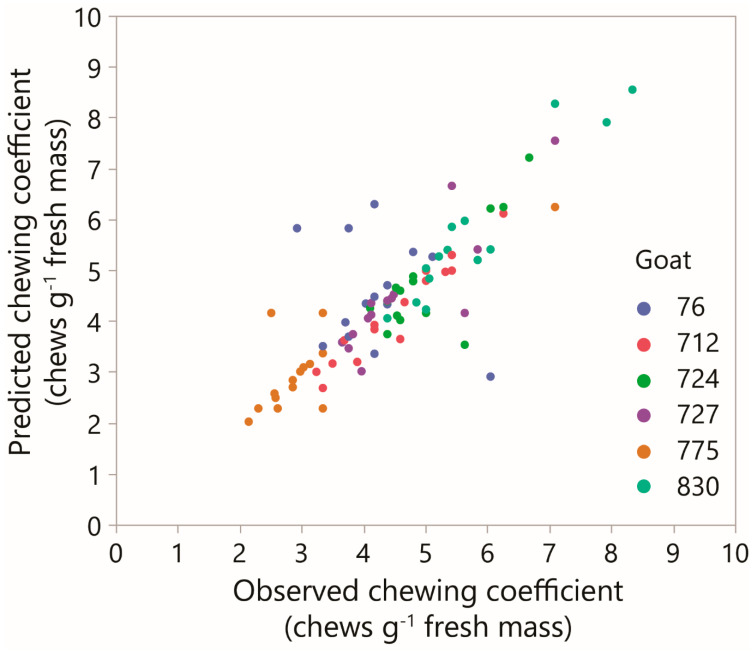
The relationship between predicted (*y*-axis) and observed (*x*-axis) chewing coefficient for Experiment 1. The six experimental animals are distinguished by color. Each point represents one foraging bout from a hand-constructed array of carob leaflets (*n* = 89).

**Figure A10:**
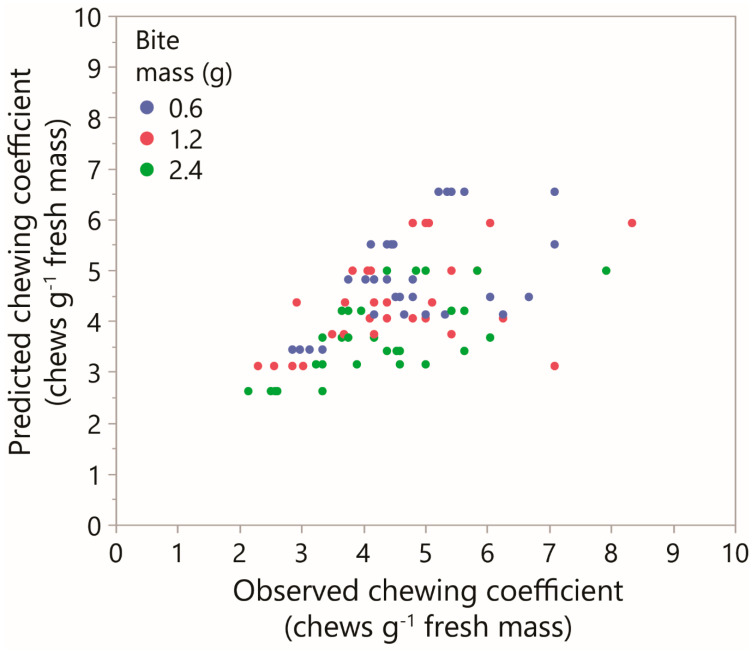
The predicted chewing coefficient (*y*-axis) versus the observed chewing coefficient (*x*-axis) after imposing a standardized mouth fill that varied with bite mass.

**Figure A11:**
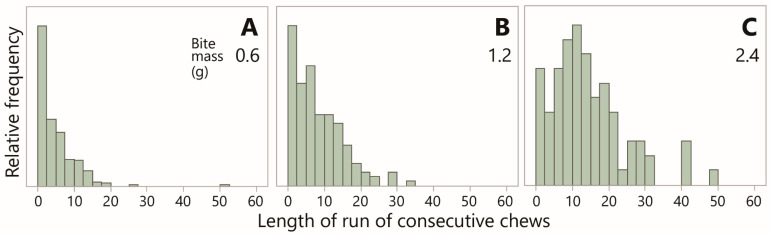
The frequency distributions of the length of runs of chews (including chew–bites) in Experiment 1, by bite mass: (**A**) 0.6 g; (**B**) 1.2 g; (**C**) 2.4 g, fresh mass. Panels are scaled equally.

## Data Availability

Data are contained within the article or Supplementary Materials.

## Supplementary Materials

The following supporting information can be downloaded at https://www.mdpi.com/article/10.3390/s26020719/s1, Supplementary Figure S1: The model plant species used in Experiments 1 and 2. Panel (A) shows one of the carob trees (*Ceratonia siliqua* L.) from which leaves were harvested. Panel (B) shows a close-up image of leaf morphology, showing a pinnate structure with six leaflets. Supplementary Figure S2: A number of the six temporary cubicles used to separate the experimental animals and control their individual intake on training and observation days of Experiment 2. Supplementary Figure S3: The relationship between total chew actions performed (*y*-axis) and (A) total intake (*x*-axis); (B) total bite actions (*x*-axis), for all test sessions in Experiment 1. Supplementary Figure S4. The relationship between total chew actions performed (*y*-axis) and total intake (*x*-axis) for all test sessions in Experiment 1 according to bite mass: (A) 0.6 g; (B) 1.2 g; and (C) 2.4 g fresh mass. Supplementary Figure S5: The frequency distributions of the inter-chew interval (log scale) in Experiment 1, by bite mass. Panels (A–C) are for consecutive chews. Panels (D–F) are for non-consecutive chews. Supplementary Figure S6: The relationship between total chew actions performed (*y*-axis) and total intake (*x*-axis) for all test sessions in Experiment 2. Supplementary Figure S7. The relationship between the total chew number (*y*-axis) and total intake (*x*-axis) in Experiment 2, by goat and satiety level. Supplementary Figure S8: The relationship between the total chew number (*y*-axis) and total intake (*x*-axis) for the combined data of Experiments 1 and 2. Supplementary Figure S9: The relationship between the proportion of all jaw movements deemed to fall in phases of mouth filling or emptying (*y*-axis) and the time threshold assumed to trigger emptying and refilling (*x*-axis). Supplementary Table S1. Basic characteristics of the experimental animals used in Experiments 1 and 2. Supplementary Table S2. Chemical analysis of the carob foliage, as predicted by NIRS analysis.

## Abbreviations

DMC = dry matter content; FMC = foliage moisture content; *m* = number of jaw movements (presumed to be chews) performed at reduced mouth fill due to an emptying phase; *n* = number of jaw movements (bites and chews) performed at reduced mouth fill due to a filling phase; NIRS = near infrared reflectance spectroscopy; *t* = threshold for the inter-jaw movement time interval above which mouth emptying and filling is presumed to have occurred; *V* = number of chew actions required to prepare any bite for swallowing; *V** = *V* that yields lowest deviation from observed sequence of chew actions

## Appendix A. Explanation of Elevated Inter-Chew-Action Intervals

Here we present a detailed explanation of the inter-chew-action intervals flagged with lower-case lettering in Figure 8 of the main article.

Note that pure bites were not precisely timestamped. To indicate their occurrence on the event-based plots without ascribing them a true time interval, pure bites were all arbitrarily designated the out-of-range interval of 0.1 s. In Panel (A), point (a) represents a chew action that was preceded by two bites, the second of which entailed numerous rapid manipulative movements of the prehensile upper lip, and was followed by pure-manipulative (non-sound-producing) and atypical jaw movements appearing to organize material in the mouth. Point (b) shows a larger interval than expected for consecutive chew actions because there were some pure manipulative movements. Point (c) is elevated because of a brief pause in jaw activity. Point (d) represents a single pure bite with elevated use of manipulative lip movements. Point (e) entails elevated use of manipulative jaw movements to organize material in the mouth.

In panel (B), points (b), (c) and (d) entailed greater use of manipulative lip movements in bite formation than point (a). The exceptionally high interval for one pair of successive chew actions (point (e)) was due to the animal being briefly distracted by the operator during which time there was a cessation of jaw activity.

In panel (C) there is reasonable separation of the inter-chew interval into two bands, with few intervals for consecutive chews falling in the range dominated by non-consecutive chews. The animal established something of a repeating pattern comprising a bite (almost exclusively pure bites) followed by two pure chews. Exceptions were point (a), which entailed a pure chew followed by the only chew–bite in the sequence, with extra time being incurred by the use of manipulative jaw movements in bite formation; and point (b), which was caused by the animal being startled momentarily (for no apparent reason).

In panel (D), point (a) was caused by the animal “freezing” momentarily and then using manipulative lip movements to perform a chew–bite; point (b) was caused by a distraction; and point (c) was the result of a brief pause.

In panel (E), points (a) and (c) are for single-bite “insertions” between two chews, the higher point (b) is for two consecutive pure bites interceding, and point (d) for a pause followed by three consecutive pure bites.

In panel (F), there is strong, separate banding of the two types of interval, with the only deviations being point (a) which entailed two consecutive pure bites and a pause, and point (b) which entailed manipulative lip movements.

## Appendix B. Elucidation of the Mechanics of the Ingestive Process Based on Acoustic Monitoring

### Appendix B.1. Evaluation of an Alternative Model of Chewing

The notion of a chewing coefficient is not used in the alternative model of chewing, although, crucially, it can be derived from it in order to convert chew counts to intake amounts. The core proposition of the model is that every “packet of intake” (bite) has a chewing requirement of a certain number (*V*) of chew actions to make it swallow-ready; *V* is not dependent on bite mass. More precisely, the constituents of every bite need to reside in the mouth for *V* chew actions in order to be reduced to the target frequency distribution of particle lengths. Conceptually, with each chew, each packet of material advances one step in its obligatory mouth residency of *V* chews. Functions of saliva mixing and associated chemical interactions (e.g., binding of tannins) may occur during the performance of the *V* chew actions. The mouth accommodates and parallel-processes multiple packets of intake, within the constraints of its capacity (*M*). This distinguishes the parameter *V* from the classic notion of chews per bite. Furthermore, *V* is assumed to be constant over the entire range of possible levels of mouth fill; a chew action will advance all material in the mouth by one step in the required breakdown process, irrespective of mouth fill.

Mouth fill is computed for each chew or chew-bite in the jaw movement stream in the following way. With each bite or chew-bite, the bite mass is added to the mouth fill at the next *V* chew actions. Once *V* chew actions have been performed on the material contained in a bite, that material is assumed to be either swallowed, or retained near the rear of the buccal cavity until flushed by the next swallow reflex. During that pre-swallow retention time, the material is not further processed by whatever chews occur, and is not included in the calculation of mouth fill.

In applying the *V*-based model to the data of Experiment 1, the observed stream of bites, chews and chew-bites is a given. The model generates the mouth fill (at each chew action) implied by a given *V*. Consider the extremes of possible *V* values. If *V* = 1, the vast majority of observed chew actions will have no corresponding simulated mouth fill. If *V* is unreasonably high, there will be an accumulation of unrealistic mouth fills, and there will be a long tail of simulated mouth fill that has no observed-chew counterpart. The predictive quality of *V* can be expressed by the number of excess chews predicted over and above the number observed, and the shortfall in the number of predicted chews relative to the number observed. Both values can be greater than zero in the same run. There is an optimal value (*V**) at which the sum of these two errors is at a minimum. The credibility of the approach lies in the magnitude of the error at *V**, and the simulated mouth fill over the course of the run–in particular, the prevalence of unreasonably high levels.

The *V*-based chewing rule was applied to the data of Experiment 1 by examining *V* values from 1 to 25, in increments of three. In general, with increasing *V*, the number of *surplus* chews starts at zero and then climbs. In parallel, the number of *missing* chews starts very high and declines to low levels (Appendix B, Figure A1). The lowest overall error is where the lines cross (minimum sum). The corresponding *V** value was determined separately for each animal; *V** ranged from 10 (goat 775, which had the lowest chewing coefficient) to 19 (goat 830, which had the highest chewing coefficient) chew actions.

At *V** for each animal, 90% of runs had an excess ≤ 5 chews, and there were no excess chews in 46 of 89 runs. In terms of deficient chews, 90% of runs had a deficit ≤ 7 chews, and there was no deficit in 65 of 89 runs. A strong linear relationship was obtained between predicted and observed total chew number (Appendix B, Figure A2).

The global pooled frequency distribution of mouth fill (Appendix B, Figure A3) was unavoidably shaped by the discrete intervals between the bite masses of Experiment 1, but indicated that 90% of values did not exceed 4.8 g, or the equivalent of two of the heaviest bite masses examined. The median mouth fill was 3 g. Both these values seem eminently reasonable. The discrete (up or down) jumps in mouth fill that occur at the two higher bite masses (1.2 and 2.4 g) make it difficult to determine whether mouth fill maintained a steady-state level. Nevertheless, of the 89 overall patterns of mouth fill examined, there were many that comprised one or more pronounced, wave-like undulations in mouth fill (Appendix B, Figure A4). In contrast, there were few that strongly suggested a steady-state of mouth fill, after allowing for sloped initial and terminal sections for filling and emptying (examples are included in Appendix B, Figure A4). Putting aside the implication of seemingly suboptimal behavior, for which we have no compelling explanation, phases of filling and emptying of the mouth (or, at least, large fluctuations in mouth fill) do not just occur at the extremities of an ingestive bout, but more widely. Furthermore, this is being purported to occur even when the foraging arena could not be more conducive to the expression of high, steady-state mouth fill.

It follows that the variable of interest that is expected to modulate the emergent chewing coefficient is the average mouth fill across all chew actions of an ingestive bout (*M*′), and this variable should bare a relationship to the observed chewing efficiency (*E*_obs_) in Experiment 1, as examined in Appendix B, Figure A5. The overall trend seems apparent, but the results per goat were mixed. For three of the goats there appears to be an inverse relationship, as expected under the *V*-based model.

Also expected under the *V*-based model is a constant number of terminal chews, i.e., chew actions following the last bite action of a run (which should equal *V**). There was only a very weak positive relationship between simulated mouth fill at last bite and number of terminal chews; variability was high (Appendix B, Figure A6).

### Appendix B.2. Simple Algebraic Formulation of the V-Based Model

Let:

B_obs_ = total number of bite actions observed

C_obs_ = total number of chew actions observed

V = number of consecutive, non-exclusive, chew actions required per bite, determined by best fit to the data.

W = bite mass (g)

M = mouth capacity, in units of mass, inferred from the frequency distribution of the mouth fill level at every chew action. For simplicity, assume M to be an integer multiple of the bite mass (W).

Chew–bites are not treated explicitly but are assumed to be equivalent to the performance of a pure chew and a pure bite.

By definition, intake (I) is given by:

I = B_obs_ ∙ W (A1)

And the chewing coefficient (E) is given by:

E = C_obs_/I = C_obs_/(B_obs_ ∙ W) (A2)

The *V*-based model enables two extremes to be defined. These are represented by the single-bite model (*sb*) and the full tank model (*ft*). The single-bite model entails the maximum possible number of chews.

C_sb_ = C_max_ = V ∙ B (A3)

It follows that:

E_sb_ = C_max_/I = (V ∙ B)/(B ∙ W) = V/W (A4)

In contrast, the full tank model (be it continuous feed or batch feed) entails the minimum possible number of chews. The number *V* ∙ *B* will be divided into the maximum number of bites that can be accommodated by the mouth, which is *M*/*W*.

C_ft_ = C_min_ = (V ∙ B ∙ W)/M (A5)

E_ft_ = C_min_/I = (V ∙ B ∙ W)/(M ∙ B ∙ W) = V/M (A6)

Although the V component of the *V*-based model is untethered to the mass ingested with each bite, mass appears in the form of mouth capacity (*M*). A finite capacity forces “queueing” of bites and the performance of chews accordingly.

### Appendix B.3. Application of the V-Based Model to Experiment 1

The variables (A1)–(A6) above were derived for the 89 runs of Experiment 1, using the animal-level values of *V**. In order to place *C*_obs_ on the range of *C*_min_ to *C*_max_ (or outside it), and likewise place *E*_obs_ on the range of *E*_sb_ to *E*_ft_, it was necessary to assume a value for *M*. This was set to 4.8 g, which was, as noted above, the 90th percentile of the frequency distribution of simulated mouth fill, for all data combined (Appendix B, Figure A3).

A comparison of *C*_obs_, *C*_min_ and *C*_max_ is shown in Appendix B, Figure A7A. The animals were clearly not operating on a single-bite basis that yields *C*_max_, and were not markedly dissimilar to the full tank basis that yields *C*_min_. Nevertheless, on closer examination, the *C*_min_ and *C*_obs_ distributions are somewhat different in shape (Appendix B, Figure A7B). Likewise, *E*_sb_ values were extremely high compared to *E*_obs_, and the more interesting comparison is between *E*_obs_ and *E*_ft_ (Appendix B, Figure A8). In effect, mouth capacity has declined from *M* to *M*′, which can then be substituted in Equation (A6) to obtain the predicted chewing coefficient as the ratio of *V** to *M*′. The relationship between predicted and observed chewing coefficient is shown in Appendix B, Figure A9. In essence, the chewing coefficient of each run is being estimated using a different underlying principle to that of dividing chew number by mass ingested. Using this approach, the issues of filling and emptying phases, or low-intake-level situations, are essentially bypassed.

### Appendix B.4. Application of the V-Based Model More Widely

A critical question is what happens when *M*′ in mass units is unknown, as would mostly be the case in field applications because bite mass is unknown. Based on Experiment 1, mean mouth fill increased with bite mass, being 3.0, 3.3 and 4.0 g at bite masses of 0.6, 1.2 and 2.4 g, respectively. If *E*_pred_ is based on these three values of *M*′ according to bite mass, the relationship between *E*_pred_ and *E*_obs_ is evident but somewhat poorer, as shown in Appendix B, Figure A10. Thus, it would be necessary to guestimate the relevant range of bite mass, and use the corresponding simulated mean mouth fill (*M*′) in conjunction with the computed *V** to obtain an estimate of the chewing coefficient (*E*) by which to convert chew counts to intake amounts.

### Appendix B.5. Chew Effectiveness and Mouth Fill

One oversimplification of the model as defined might be the presumption of a zero-intercept, linear relationship between the amount of herbage processing achieved in a chew and mouth fill. A case could be made that the amount of processing achieved per “packet” of herbage in the mouth depends on mouth fill, being suppressed at the low and high ends of the scale. Consequently, the amount of herbage processing achieved in a chew may well rise more slowly at low mouth fill, and also as fill approaches capacity. Although that complicates the computations, the underlying approach remains the same.

### Appendix B.6. Explanations Under the V-Based Model

The “explanation” for the effect of bite mass on chewing coefficient under the *V*-based model is an increase in average mouth fill with increasing bite mass. More herbage gets processed with each chew (this makes lower mouth fills seem suboptimal, for which we have no compelling explanation). The “explanation” for the effect of intake amount on chewing coefficient under the *V*-based model is that low intake level resulted in mean mouth fill being low, and substantially so at the lowest intake level; low mean mouth fill is associated with higher chewing coefficient (*E*) (Appendix B, Figure A5).

### Appendix B.7. Qualitative Inferences from Chew and Bite Sequencing

In the algebraic development of the *V*-based model above (Appendix B.2) the extremes of possible chewing effort corresponded to single-bite versus tank-based sequencing of jaw movements. Some qualitative inferences regarding their relevance can be drawn from the chew–intake staircase diagram shown in Figure 11 of the main article, that compared results for the five levels of intake, all based on the same bite mass (the lowest tested being 0.6 g). The following broad features emerged: (i) for the most part, and within animal, the different total amounts consumed generated similar and partially overlapping pathways; (ii) the overall slope of the staircase-like pathway can vary widely among animals; (iii) almost invariably, staircases show a fine interleaving of chews and bites; and (iv) staircases are generally terminated with an atypically long run of chews that are of comparable length across intake levels and animals (perhaps suggestive of the *V*-based model). If the ingestive strategy of the animal was batch processing, we would expect longer runs of bites, and a greater tendency for alternation between runs of bites for the filling phase, and runs of chews for the processing phase. Thus the smoothness of the chew–intake staircase diagram at the lowest tested bite mass leans strongly against a batch-feed tank model.

The aforementioned smoothness of the chew–intake staircase diagram for low bite mass could theoretically be consistent with the single-bite processing model. However, a different feature of the same staircase diagram weighs strongly against the single-bite processing model, and that is the universal presence of an atypically long run of chews after the last bite action (terminal chews). In the single-bite model, no more chewing is expected at the end than at any other stage in the depletion process. However, consistent terminal chewing does lean strongly in favor of a tank-based model, with the amount of herbage maintained in the mouth (representing a “full tank”) being commensurate with the amount of terminal chewing that was required to clear it. The fine interleaving of chews and bites along the main stretch of the staircase diagram strongly supports the continuous-feed tank model.

### Appendix B.8. Runs Analysis

Qualitative inferences can also be made from runs analysis. We tested if the frequency distribution of runs of bites differed between bite mass treatments of Experiment 1. For the purposes of this analysis, chew–bites were treated as bites in that they continued an existing bite run, and initiated a new bite run when following a pure chew. Appendix B, Table A1 shows the frequency distribution of run length, ranging from one to six consecutive bites. A total of 880 bite actions (pure bites or chew–bites) were performed in the course of the entire experiment, of which 631 (72%) were performed as singletons. Notably, singletons also dominated results for each bite mass. Doubles occurred even at a bite mass of 2.4 g, accounting for 18% of all bite actions, indicating again that this bite mass was not excessive relative to the volitional capacity of the mouth. At a bite mass of 1.2 g, triples and quadruples occurred, but infrequently, and at a bite mass of 0.6 g, a quintuple and a sextuple were recorded. But, at a bite mass of 0.6 g, there was no major shift from singletons to longer runs that would be expected under the batch tank model.

We likewise examined if the frequency distribution of runs of chews differed between bite mass treatments. For the purposes of this analysis, chew–bites were treated as chews in continuing an existing chew run, or in initiating a new chew run when following a pure bite. There was a strong shift in the frequency distribution towards longer runs of chews as bite mass increased (Appendix B, Figure A11); the median length of a chew run was 3, 7 and 12 for bite masses of 0.6, 1.2 and 2.4 g, respectively. This would not be expected if the underlying strategy was the batch tank model, but is compatible with both the single bite and the continuous-feed tank models.

**Table A1:** The frequency distribution of the length of bite runs comprising pure bites and/or chew–bites, for a global analysis of Experiment 1, and for each bite mass separately.

	Bite Mass (g Fresh Mass)											
	All			0.6			1.2			2.4		
Run Length	*n* Runs	*n* Bites	Prop. of Bites	*n* Runs	*n* Bites	Prop. of Bites	*n* Runs	*n* Bites	Prop. of Bites	*n* Runs	*n* Bites	Prop. of Bites
Singleton	631	631	0.72	239	239	0.67	119	119	0.61	75	75	0.82
Double	82	164	0.19	27	54	0.15	33	66	0.34	8	16	0.18
Triple	14	42	0.05	11	33	0.09	2	6	0.03			
Quadruple	8	32	0.04	6	24	0.07	1	4	0.02			
Quintuple	1	5	0.01	1	5	0.01						
Sextuple	1	6	0.01									
All		880			355			195			91	

### Appendix B.9. In Summary

The inferences made regarding the plausibility of alternative models of herbage processing in the mouth are summarized in Appendix B, Table A2. It seems clear that the strategy is not based on the batch-feed tank model, which would be expected to manifest, at least at the higher levels of intake, as distinguishable loading and processing phases. The batch model was hard to reconcile with the fine interleaving of chews and bites at the lowest bite mass tested. It seems clear also that the strategy is not bite based either, which would be expected to manifest as the typical inter-bite quantity of chewing following the last bite, and not the pronounced terminal chewing that was evident. The continuous-feed tank model was compatible with all aspects of the observed results.

In the mass-based interpretation of the chewing coefficient, the animal is assumed to maintain significant mouth loading (“full tank”) with “continuous feed”–meaning that the animal interleaves bites, predominantly occurring as singletons across bite masses, with a number of chews commensurate with the bite mass. The last bite is followed by an atypically long run of chews to clear the mouth loading. This model was consistent both with expectations on evolutionary grounds, as touched on in the Introduction of the main paper, and with the empirically-observed stability in chewing coefficient. According to the *V*-based interpretation of chewing coefficient, a similar logic applies. Whatever pattern was traced out by mouth fill is reflected in the mean value, which is then used to derive the chewing coefficient. Here too, the last bite is followed by an atypically long run of chews to clear the mouth loading.

**Table A2:** A summary of inferences made regarding the alternative models of herbage processing in the mouth based on results observed in Experiment 1.

	Model of Herbage Processing		
Result Observed	Single Bite	Batch-Feed Tank	Continuous-Feed Tank
Different intakes generated similar trajectories	Expected	Less likely	Expected
Fine interleaving of chews and bites at lowest bite mass	Expected	Incompatible	Expected
Extended terminal chewing	Incompatible	Unlikely	Expected
Dominance of bite singletons across bite masses	Expected	Incompatible	Expected
Longer chew runs with increasing bite mass	Compatible	Incompatible	Expected
