# Supplementary material for: From Chew Counts to Intake Amounts: An Evaluation of Acoustic Sensing in Browsing Goats
Source: Sensors (Basel). 2026 Jan 21;26(2):719. doi: 10.3390/s26020719 (PMC12845949; doi:10.3390/s26020719)
Supplement: Supplementary file 1 [file sensors-26-00719-s001.zip › sensors-4067586-supplementary.pdf]

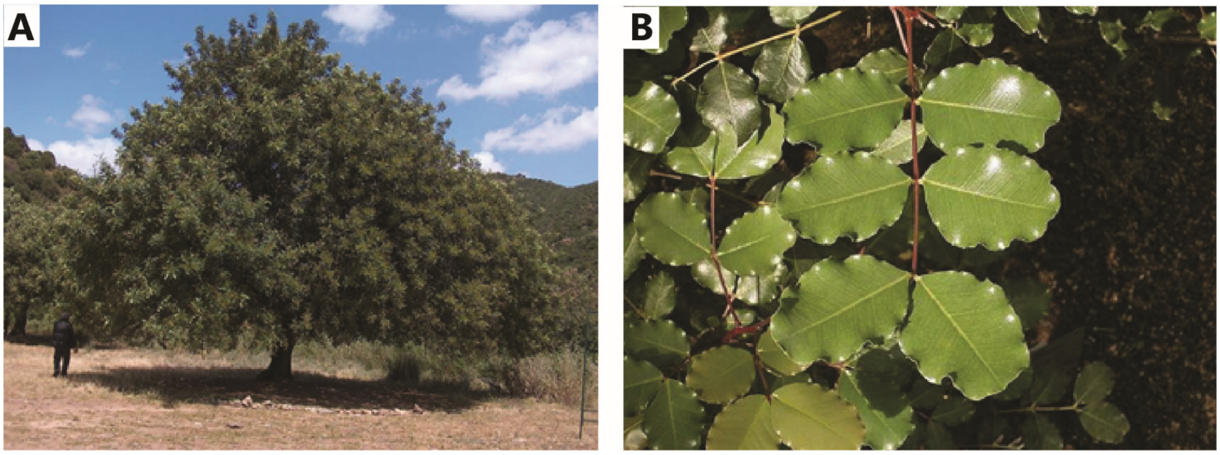

Supplementary Figure S1. The model plant species used in Experiments 1 and 2. Panel (A) shows one of the carob trees (*Ceratonia siliqua* L.) from which leaves were harvested. Panel (B) shows a close-up image of leaf morphology showing pinnate structure with six leaflets (leaflet number is variable). The fresh mass of one leaflet was approximately 0.6–1.0 g. Photos courtesy of Shilo Navon.

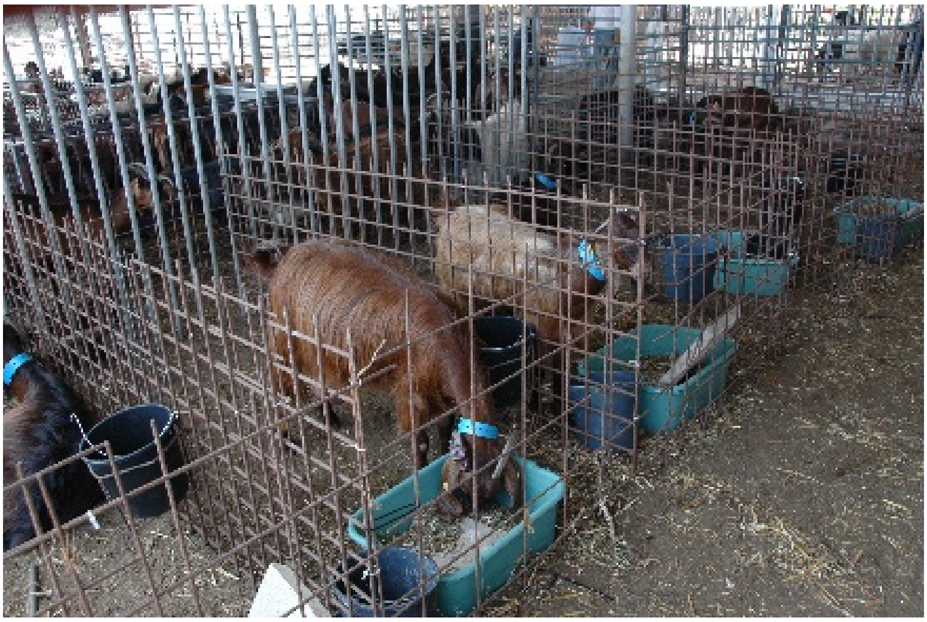

Supplementary Figure S2. A number of the six temporary cubicles used to separate the experimental animals and control their individual intake, on training and observation days of Experiment 2. Photo courtesy of Shilo Navon.

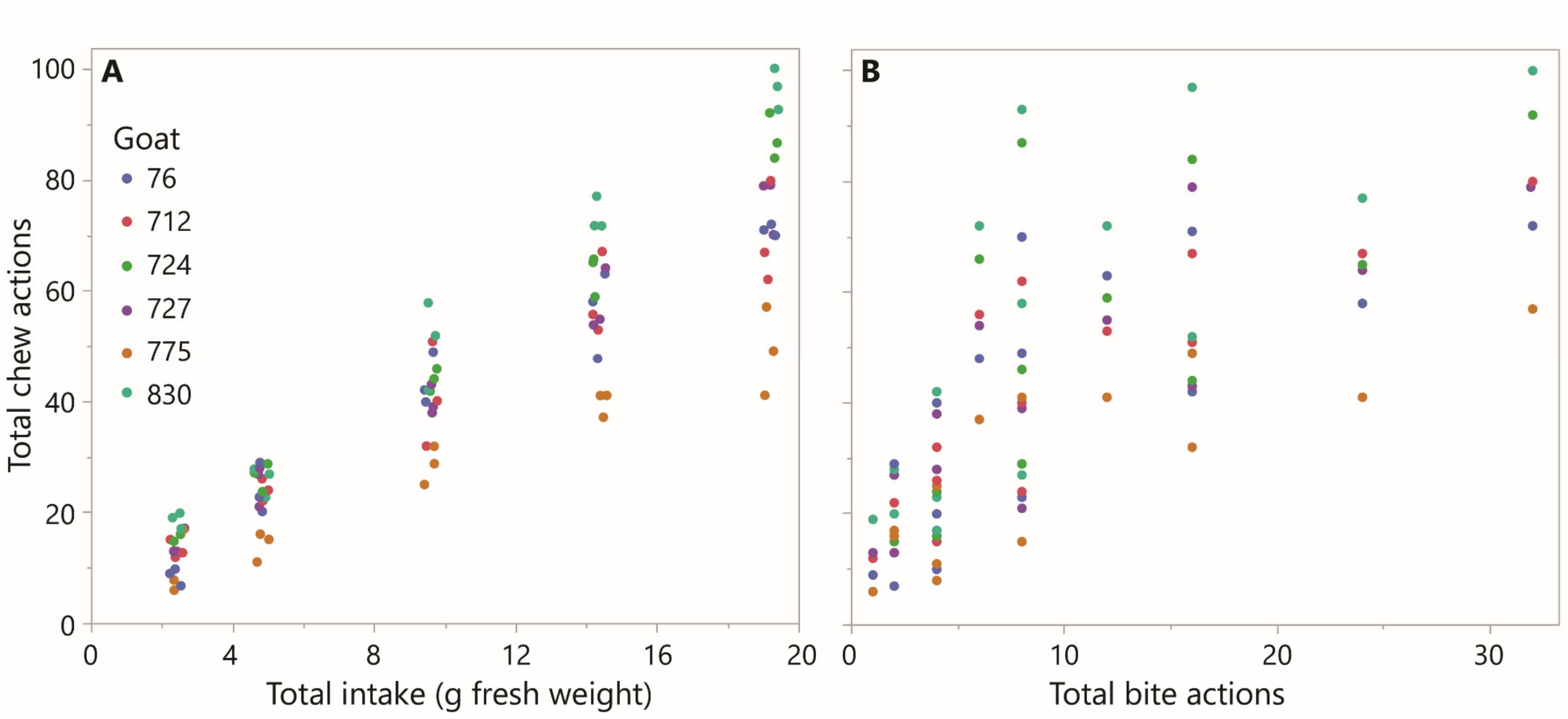

Supplementary Figure S3. The relationship between total chew actions performed (*y*-axis) and (**A**) total intake (*x*-axis); (**B**) total bite actions (*x*-axis), for all test sessions in Experiment 1 (*n* = 89). The six experimental animals are distinguished by color. Points in panel (**A**) are jittered to reduce overlap. Some degree of segregation by color in (**A**) suggests that the individual animal is a major source of variation.

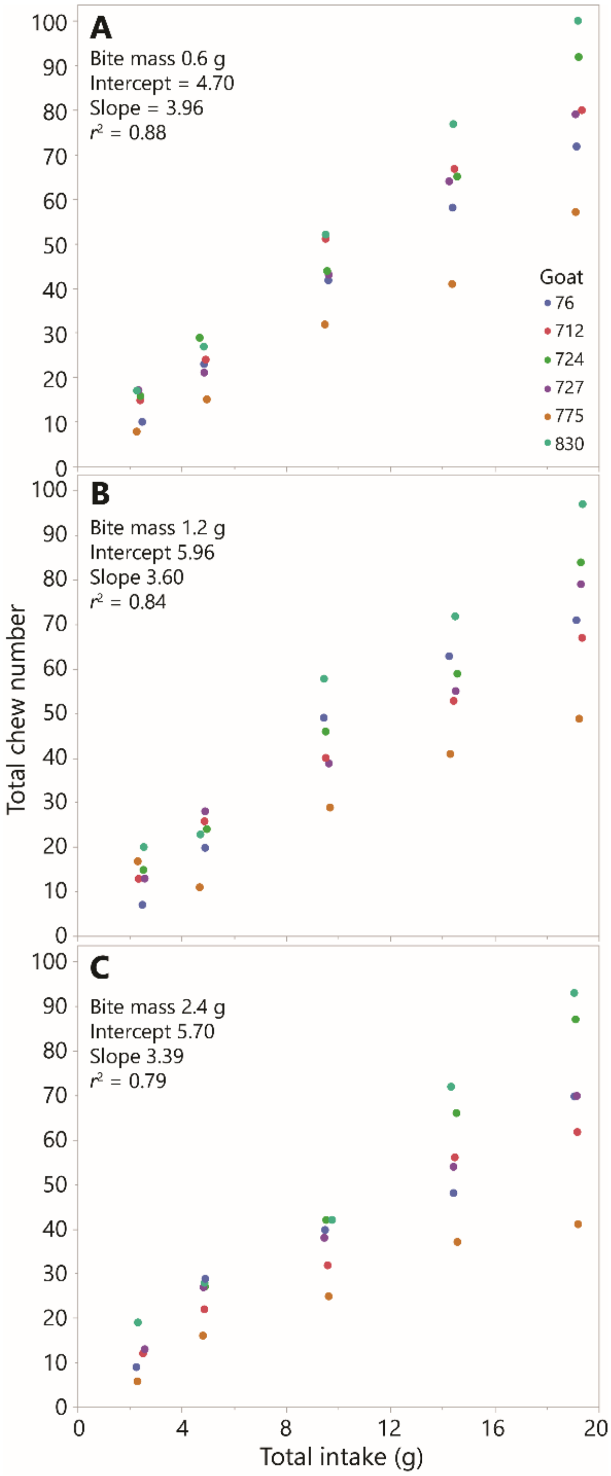

Supplementary Figure S4. The relationship between total chew actions performed (*y*-axis) and total intake (*x*-axis) for all test sessions in Experiment 1 (*n* = 89) according to bite mass: (**A**) 0.6 g; (**B**) 1.2 g; and (**C**) 2.4 g fresh mass. The six experimental animals are distinguished by color. Points are jittered to reduce overlap.

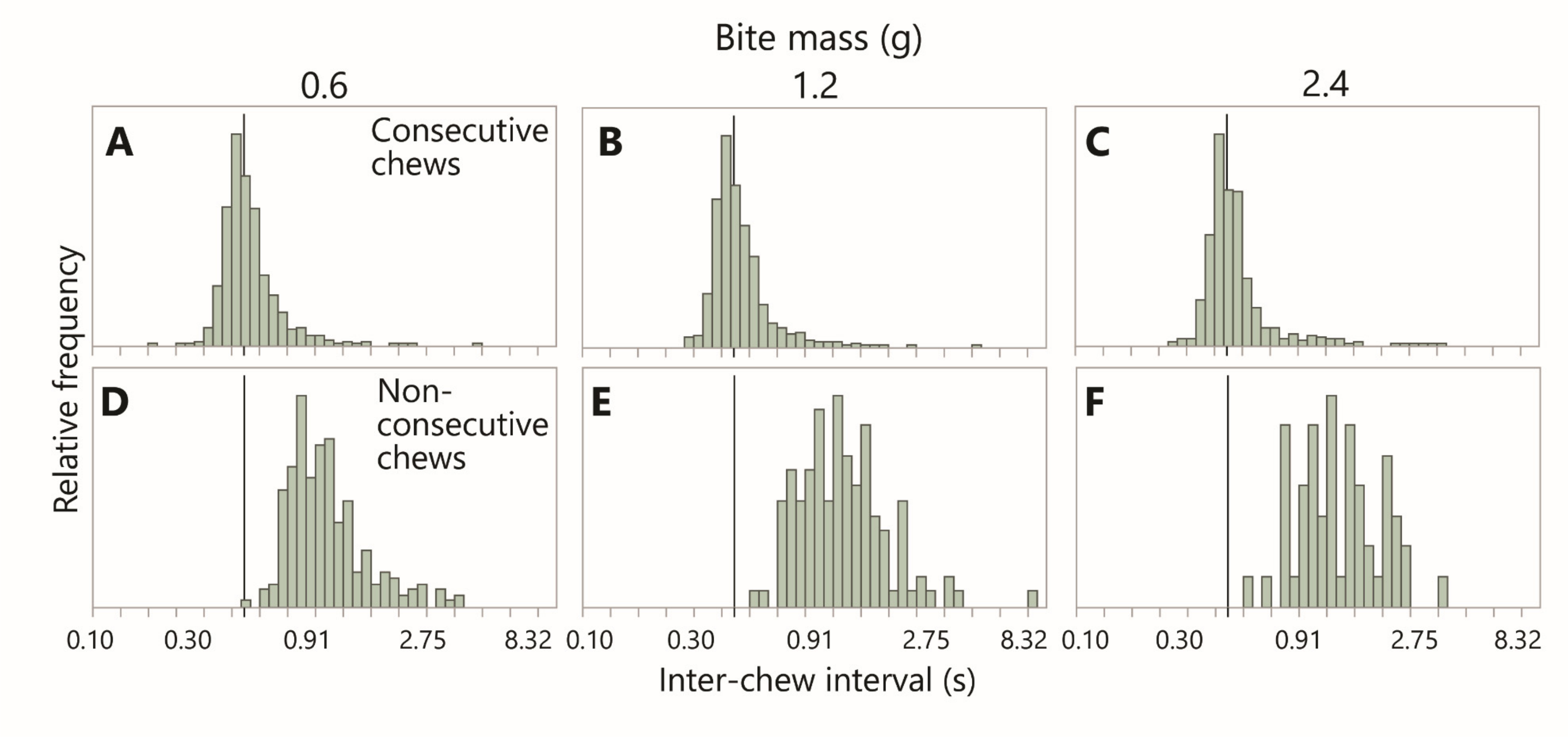

Supplementary Figure S5. The frequency distributions of the inter-chew interval (log scale) in Experiment 1, by bite mass. Panels (**A–C**) are for consecutive chews. Panels (**D–F**) are for non-consecutive chews. Panels are scaled equally. The vertical reference lines are at 0.45 s.

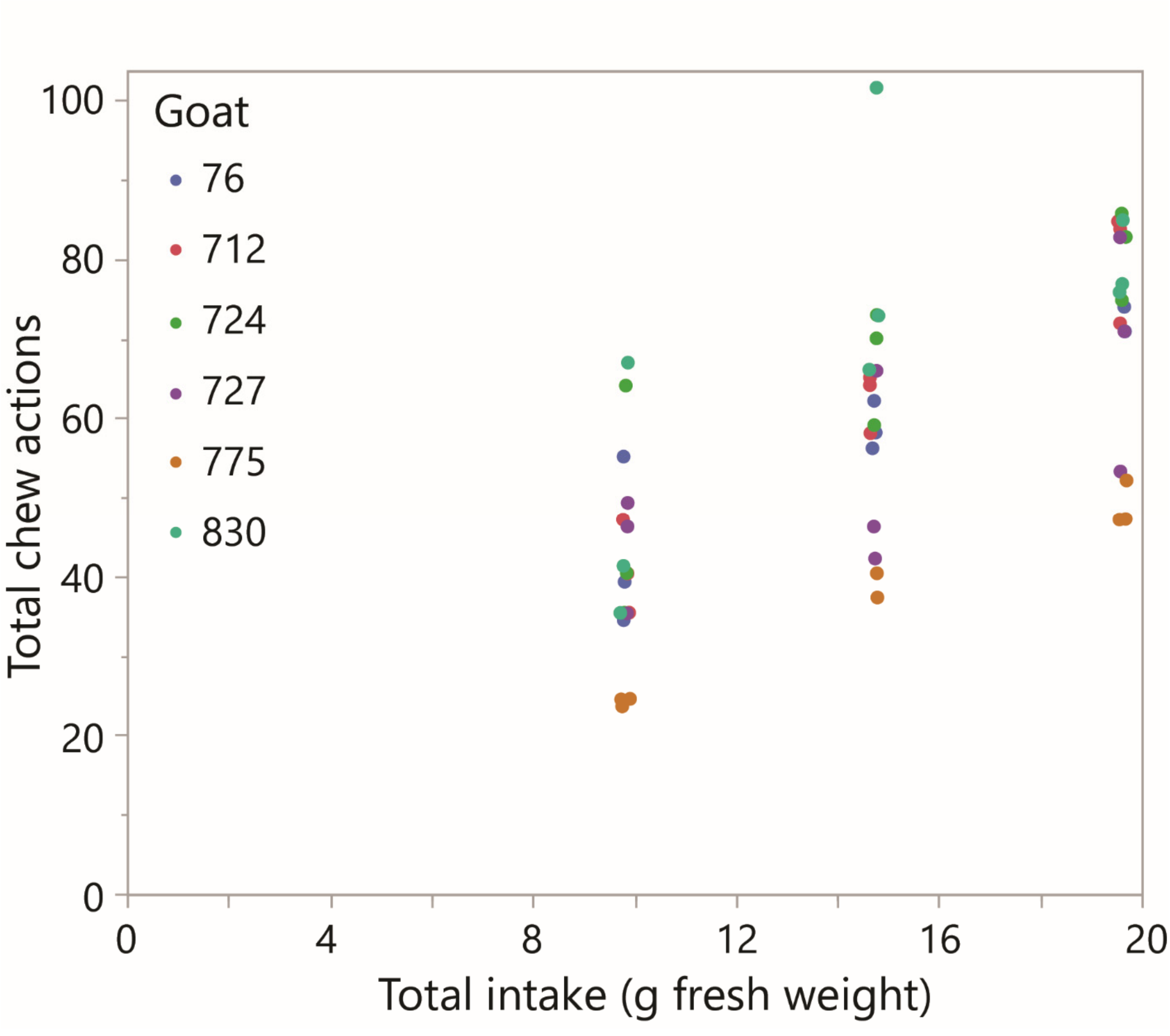

Supplementary Figure S6. The relationship between total chew actions performed (*y*-axis) and total intake (*x*-axis) for all test sessions in Experiment 2. The six experimental animals are distinguished by color. Points are jittered to reduce overlap. The video recordings revealed nothing exceptional to justify exclusion of the highest chew count recorded (goat 724; 14.4 g intake).

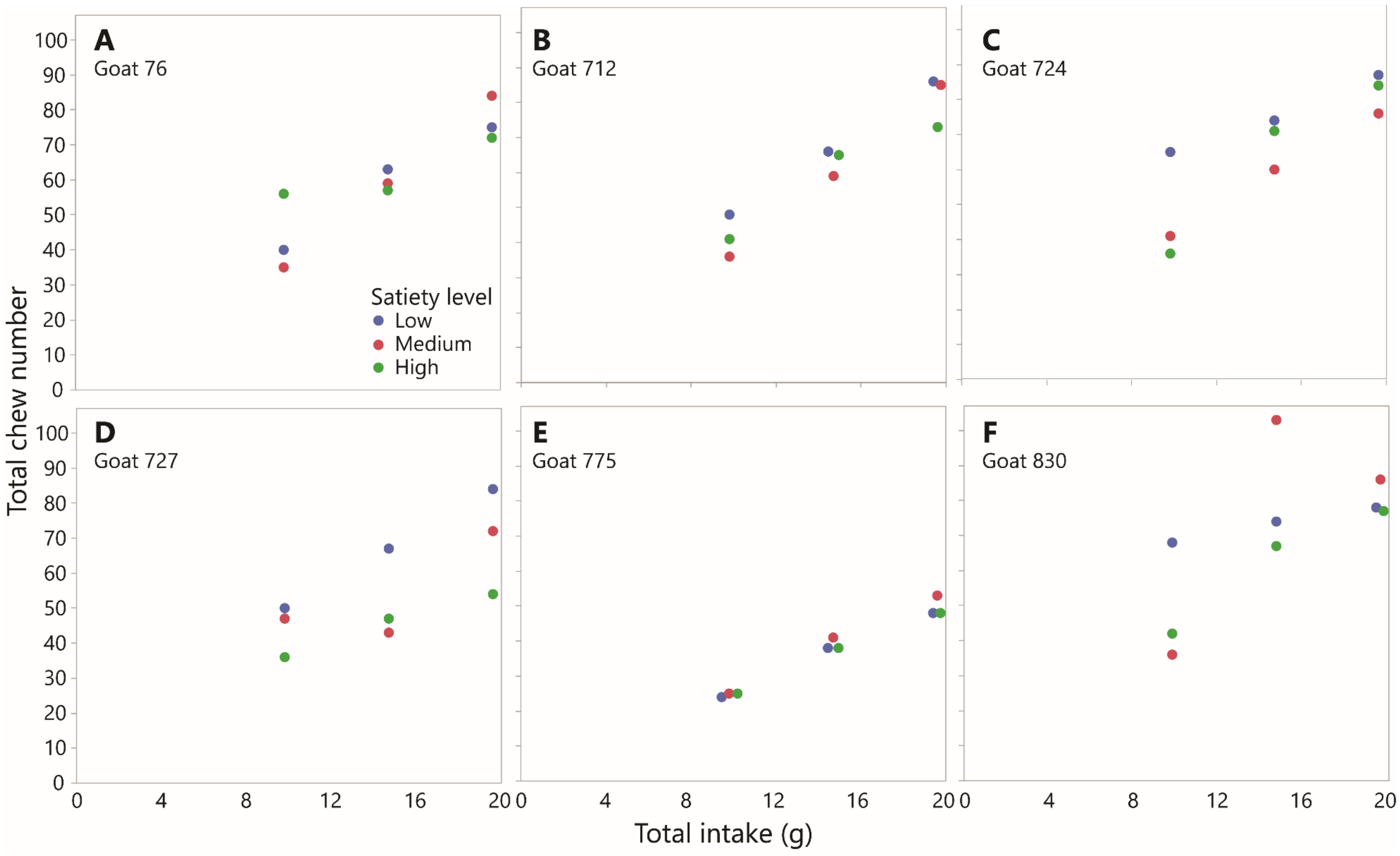

Supplementary Figure S7. The relationship between total chew number (*y*-axis) and total intake (*x*-axis) in Experiment 2, by goat and satiety level. Panels are scaled equally. Points are jittered to reduce overlap.

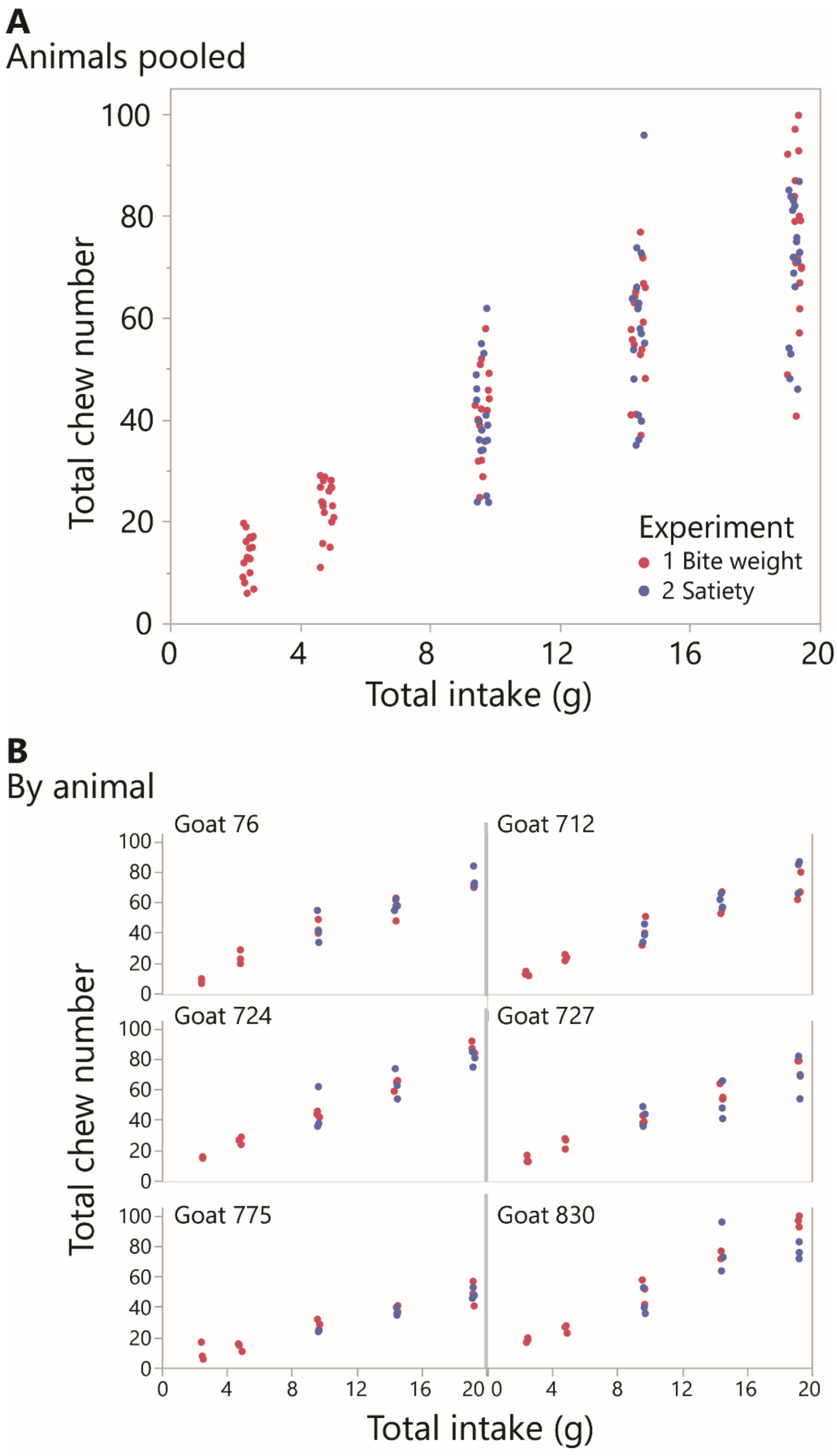

Supplementary Figure S8. The relationship between total chew number (*y*-axis) and total intake (*x*-axis) for the combined data of Experiments 1 and 2: **(A)** for all animals pooled; **(B)** for each animal separately. Points are jittered to reduce overlap.

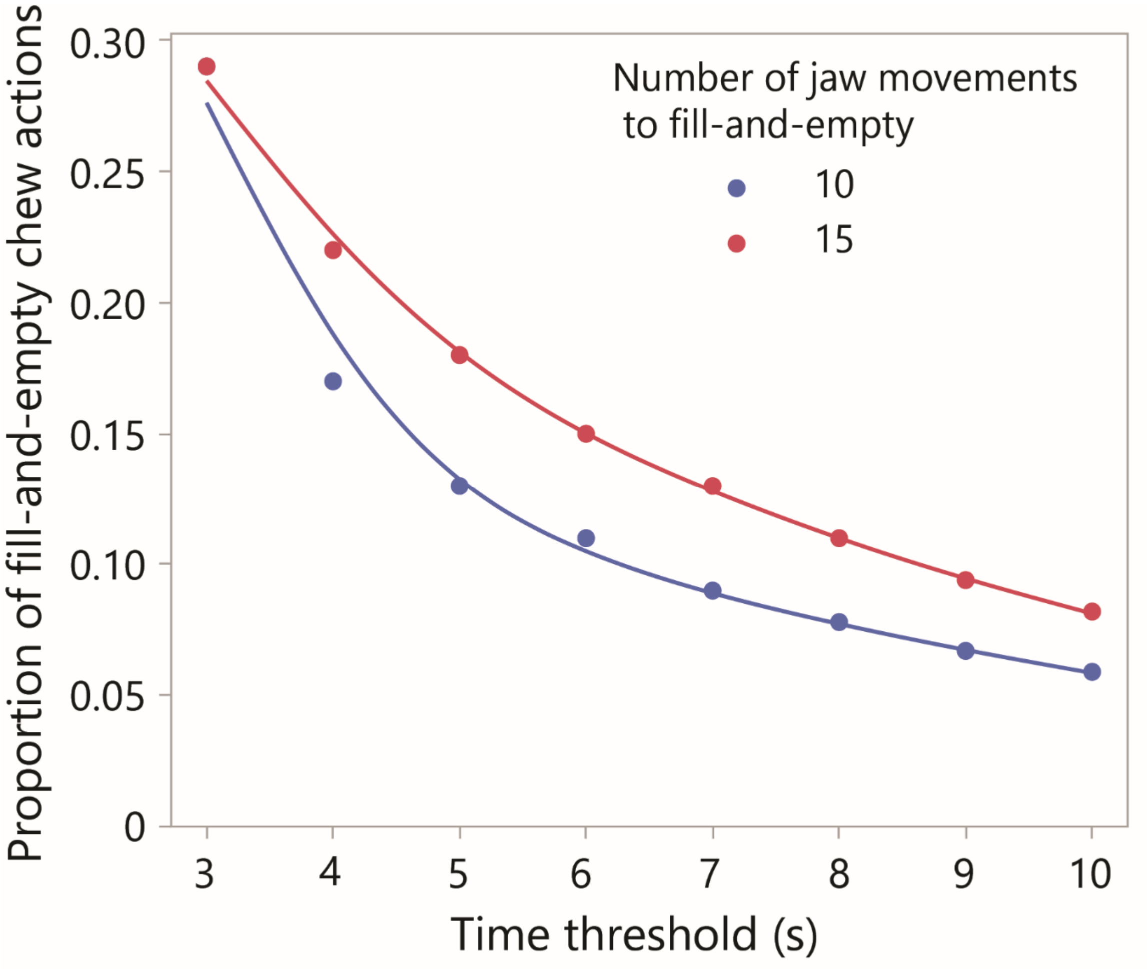

Supplementary Figure S9. The relationship between the proportion of all jaw movements deemed to fall in phases of mouth filling or emptying (*y*-axis) and the time threshold assumed to trigger emptying-and-refilling (*x*-axis). Two values (10 and 15) were tested for the total number of jaw movements in one emptying-and-filling cycle. The analysis was based on the acoustically-derived timelines of jaw activity reported in Ungar and Horn [7], comprising 12 daily foraging routes of goat herds in a hilly Mediterranean landscape with substantial cover of woody vegetation.

Supplementary Table S1. Basic characteristics of the experimental animals used in Experiments 1 and 2. All animals were primi- or multiparous dairy goats of the Damascus (Shami) breed. Age is at the start of Experiment 1 (24 April 2012); live weight is on 26 January 2012; last kidding date, litter size and average milk yield of current lactation are as of 7 June 2012.

Goat	Date of birth	Age (yr)	Live weight (kg)	Last kidding date	Litter size	Average milk yield (L d^{-1})
775	17 January 2006	6.3	69	21 January 2012	1	1.40
76	28 January 2008	4.2	64	22 January 2012	2	1.25
712	18 April 2009	3.0	59	20 January 2012	1	0.90
830	16 April 2010	2.0	54	21 January 2012	1	0.43
724	8 January 2011	1.3	44	6 March 2012	1	0.87
727	14 January 2011	1.3	65	20 January 2012	2	0.80

Supplementary Table S2. Chemical analysis of the carob foliage, as predicted by NIRS analysis. All values are percentages, on a dry-matter basis.

Component	Experiment 1	Experiment 2
	%	
Ash	4.6	6.3
Neutral detergent fiber	34.3	33.4
Acid detergent fiber	26.1	23.4
Acid detergent lignin	12.0	11.1
Crude protein	9.5	12.2
Digestibility	32.2	45.5
PEG-binding tannins	18.7	12.1
Condensed tannins	16.5	11.2
Total phenols	19.4	15.4
